# Determinants of life satisfaction among migrants in South Africa: an analysis of the GCRO’s quality of life survey (2009–2021)

**DOI:** 10.1186/s12889-023-16868-1

**Published:** 2023-10-18

**Authors:** Monica Ewomazino Akokuwebe, Salmon Likoko, Godswill N Osuafor, Erhabor Sunday Idemudia

**Affiliations:** 1 Faculty of Humanities, North-West University, Mafikeng, 2735 South Africa; 2 Statistics South Africa, ISIbalo House, Koch Street, Salvokop, Pretoria South Africa; 3 Department of Population Studies and Demography, North-West University, Mafikeng, 2735 South Africa

**Keywords:** Life satisfaction, South Africa, Internal migrants, International migrants

## Abstract

**Background:**

Migrant populations in any country are a vulnerable group, and psycho-demographic research measuring life satisfaction has been used to assess migrants’ well-being in developed and developing countries. However, South Africa, with its high influx of migrant populations, has investigated these topical concerns from the perspective of xenophobia, with mixed findings. However, no, or very few studies have examined life satisfaction among migrants in South Africa. This study, therefore, extends previous literature by examining the determinants of life satisfaction among South Africa’s internal and international migrant populations.

**Methods:**

We conducted a cross-sectional study from the 2009 to 2021 Gauteng City-Region Observatory (GCRO) Quality of Life (QoL) surveys among migrant populations in two ways: a full sample and a gender-stratified sample. A sample of male and female migrants ranging from 15 to 49 years of age were recruited into this study. Cantril’s Self-Anchoring Ladder Life Satisfaction scale captured their life satisfaction alongside relevant social demographic factors. Descriptive statistics were applied for the data analysis of the demographic factors. Bivariate and multivariate logistics regression analyses were conducted to assess the associations and the predictive factors of life satisfaction among migrants, both internal and international.

**Results:**

The key findings were the gender distribution of life satisfaction, showing that more international (male – 66.0% and female – 67.1%) migrants reported having a thriving life satisfaction than internal migrants (male – 61.7% and female – 61.5%). Findings from the Pearson correlation coefficient revealed a significant association between the individual, household, and community factors by migrant status (ρ < 0.05). However, the probit coefficients revealed that individual factors (age 48^+^: AOR = 2.18, 95% CI: 1.13, 3.23, and secondary/higher education: AOR = 1.1., 95% CI: 0.01, 1.19) and household factors (two persons living in households (H/H): AOR = 1.05, 95% CI: 0.50, 1.10), and community factors (international migrant status: AOR = 2.12, 95% CI: 0.08, 2.16) significantly increase the prediction of higher odds of life satisfaction by gender among migrants. The ordered logit coefficients also showed that individual factors (middle and high income and having health insurance) and household factors (receiving SASSA social grant) predicted the highest life satisfaction among migrants (internal and international).

**Conclusion:**

We found substantial evidence that individual-, household-, and community-level factors were associated with life satisfaction among migrants. In particular, the pattern of life satisfaction varied slightly between male and female migrants, as well as with migrant status in South Africa. These findings collectively may provide helpful information for policymakers and practitioners to optimise interventions for migrant populations to improve their life satisfaction. Evidence from this study also calls on the government of South Africa to begin tracking the life satisfaction of its nationals, whether migrants or not.

**Supplementary Information:**

The online version contains supplementary material available at 10.1186/s12889-023-16868-1.

## Introduction

South Africa is one of the top destination countries for migrants in Africa, with a migrant population of 2,137,519 (28%), as there are migration routes in and out of South Africa, most especially from neighbouring countries such as Zimbabwe and Mozambique [1]. Given its advanced economy and relative political stability, South Africa has also experienced high volumes of immigration in recent years, attracting migrants, asylum seekers, and refugees from within and outside southern Africa. As a result, migration to and within South Africa is dynamic and diversified, even though migrants face a risky labour market and many forms of discrimination [2, 3]. Intra-regional labour migration is also well-established in South Africa. Many people migrate from countries such as Eswatini, Lesotho, Malawi, and Zimbabwe to work in South Africa and Botswana. International migration is enhanced by migration policies to support various forms of migration, including visits and vacations, study, and the movement of skilled labour [4, 5]. Recent studies have revealed that the number of international migrants in South Africa has increased from 2 million in 2010 to over 4 million in 2019, but then declined to nearly 2.9 million in mid-2020. This amounts to roughly 4.8% of South Africa’s total population. Of these migrants, 43.1% were female, 11.1% were 19 and younger, and 7.1% were 65 and older [5]. However, data on levels of internal migration in Africa are limited, with South Africa and Zambia revealing greater levels of internal migration among countries in the southern African region [5].

Significantly, the economic footing of South and southern Africa was built on internal and cross-border workforce migration, with migrant remittances providing primary upkeep to immediate family, households, and communities [5]. These interconnections between urban and rural areas have persisted post-apartheid. However, in present-day South Africa, the prevalence of internal migration is labour-related, as the numbers far exceed those of cross-border movement. The most recent population census indicates that 5% of the population had moved within the country in the five years preceding the census, compared with 1% of the population who migrated from outside the country’s borders, as documented [5]. One of the primary reasons for migration is to enjoy better employment and earnings prospects, as the typical movement of people is from unindustrialised to industrialised nations at the global level, and internally from rural to urban areas, or from poorer areas to more affluent ones, and this description better explains the life satisfaction of migrants. The South African Census Data [6] showed that inter-provincial migrants were more likely to be employed and fared better in the labour market in the destination provinces [5].

Moreover, it is worth mentioning that the relationship between migration and life satisfaction is complex and repeatedly presents methodological challenges. The life satisfaction of internal migrants may differ from that of international migrants before migrating, both at the time of migration, and after, making it challenging to disentangle selection effects and the direct effects of migration on life satisfaction [7, 8]. As a result of several comparisons between international and internal migrants, while identifying the gaps, these studies are inadequate to identify the reasons for choice of comparisons as a group or as separate groups. Furthermore, migration itself may produce life satisfaction changes at key stages in one’s life course, often ascribed to the burden associated with migration. From the psycho-demographic perspective, life satisfaction is an individual assessment process in which persons liken their perceived status quo to their expectations and opportunities of the situation, either in ideal or in reference circumstances [9, 10]. As far as the migrant population is concerned, existing studies have identified three dimensions that are openly linked to migrants’ prejudiced valuation process, explicitly as cultural, social, and economic integration [11, 12]. In terms of economic integration, good living conditions depend not only on the affluence and resources of an individual but also on other factors that collectively influence life satisfaction. This implies that the quality of life of a person is positively associated with the life satisfaction of the individual migrant involved. Those international migrants migrating to the host country will likely favour a more encompassing system of possessing relatively fewer resources and relying heavily on publicly-provided facilities [13, 14].

In industrialised countries, factors such as household and personal income, marital status, educational attainment, health, age, and gender have been recognised as essential indicators of life satisfaction [15]. According to Agyekum [16] and Owusu et al. [17], their studies revealed that families with a high household income had better life satisfaction, while Meyer et al. [4] showed that life satisfaction levels are increased with marriage and childbirth, but reduced with marital separation, starting a new job, job loss, and relocating [17]. However, similar findings on associated factors have been reported in studies from sub-Saharan Africa, particularly in Malawi, Ghana, South Africa, Nigeria, and Ethiopia [17]. In the South African context, a few studies have been documented to show factors such as belonging to a religion, experiences of migration, high income, higher educational level, social capital, tobacco use, being in the lower class, residing in the southern geographical region, job security, and being married, were related to life satisfaction [18, 19]. In contrast, studies conducted in South Africa provided substantive preliminary inferences, yet have recommended the need for more research into the factors connected with life satisfaction. Besides, most of these studies have concentrated on only one aspect of life satisfaction [19]. Also, Collinson et al. [20] and Angelini et al. [21] have operationalised life satisfaction in terms of individuals’ living standards rather than how they feel about their overall life satisfaction.

Nevertheless, Ginsburg et al. [22] and Ginsburg et al. [23], with the most recent studies in South Africa, have measured life satisfaction as the extent to which persons have feelings about their overall life, although both studies relied on a nationally representative sample of men and women, as well as methodological limitations worth mentioning. The dataset that Refaeli et al. [24] used in their analysis is now quite old, as it was collected in 2005–2008. Switek’s study [25] focused only on older adult respondents who were aged 50 years and above. Given these limitations and the unrelenting requirement to produce additional contemporary findings, specifically as a result of continuing background factors ranging from inadequate rights to use basic amenities [26], a high incidence of chronic ailments [25], joblessness mainly among the youth [27], limited access to healthcare [28], poor quality education, and a high burden of poverty [29], as these may threaten one’s life satisfaction, this study used the Gauteng City-Region Observatory’s Quality of Life Survey conducted in South Africa (2009–2021) to explore the factors associated with life satisfaction among migrants in South Africa [30–35].

Our study goes further by examining these factors closely from a gendered perspective.

To the best of the authors’ knowledge, there is a dearth of such studies conducted on life satisfaction among the migrant population (involving both internal and international migrants) with associated factors (individual-level, household-level and community-level) in South Africa. The justification for engaging in a gendered perspective in this study is that it will provide more meaningful information into the associated factors of both male and female life satisfaction, as there are disparities in their social norms and biological features regarding gender [17, 27]. Studies showed outcomes where socio-political, employment-related, and education-related variables were found to be more significantly associated with male life satisfaction, while female life satisfaction was associated with factors such as social relationships and status of their marital union [23, 27]. So, it is tenable to anticipate that such exceptional differences will occur in our study outcomes.

This study, therefore, extends previous literature by examining the determinants of life satisfaction among South Africa’s internal and international migrant populations, by adapting a psycho-demographic perspective, which is the gap this study intends to fill. The specific objectives of this study, therefore, attempt to extend the empirical evidence on socio-demographic factors (individual, household, and community) and life satisfaction by using a population of migrants (internal and international) in South Africa to:- measure the gender distribution of life satisfaction by migrant status, assess the bivariate relationship between life satisfaction and socio-demographic factors (individual, household, and community) by migration status, and explore the predictors of life satisfaction by gender. Hence, the study findings will be helpful for policymakers, researchers, and practitioners, in designing gender-specific interventions and services to improve the life satisfaction of migrants, including both male and female populations, in South Africa.

### Theoretical perspective

This study is anchored on Life Satisfaction Theory and Psychology research, in which has been discussed intensively: the bottom-up and top-down theory [36, 37], which is used to explain the associations between pyscho-demographic determinants and life satisfaction. The bottom-up theories of life satisfaction, proposed by Diener [36], are based on the notion that, in total, life satisfaction is the sum of its parts; that is, self-reports of life satisfaction act as a weighted average of satisfaction with different domains of life. Also, the top-down theory sees general life satisfaction or specific areas of life satisfaction due to personality and other constant characteristics or conditions. This implies that life satisfaction is determined by traits disposal, manifesting in somewhat invariable rational and emotional conditions, ensuring individuals display stable behavioural patterns [38, 39]. However, this study utilized bottom-up theory in order to explain relationships relating to pyscho-demographic determinants and life satisfaction.

Specifically, several factors are found to influence life satisfaction, including sociodemographic factors (such as household, family, age, gender, education, health, job, income, and occupation) and psychosocial factors (such as psychological characteristics, lifestyle, participation in vacation activities) [40, 41]. Some studies have mentioned other factors that connect subjective well-being and life satisfaction, which are important demographic factors (such as gender, age, marital status, income, and education) and psychosocial factors (such as health and illness, functional ability, activity level, and social relationships). Some other studies have explored and supported the bottom-up theories in explaining psycho-demographic studies [41, 42], showing different levels of satisfaction, which may significantly predict overall life satisfaction. Therefore, we would expect to see how determinants can explain the inconsistencies arising from life satisfaction of South African migrants if an integrated account of life satisfaction is supported.

## Methods

### Study area

South Africa, officially the Republic of South Africa (RSA), is the southernmost country in Africa, with over 60 million people, and it covers an area of 1,221,037 square kilometres (471,445 square miles). The country surrounds Lesotho and is bordered by Namibia, Botswana, Zimbabwe, Mozambique, Eswatini, and the Atlantic and Indian oceans. The country has three capital cities, with the executive, judicial and legislative tiers of government established in Pretoria, Bloemfontein, and Cape Town, respectively [43]. About 81% of the population are Black South Africans, and the remaining population consists of White, Asian/Indian, Coloured and others, with the Zulu tribe being the most dominant ethnic group. The country’s population’s religious composition is 80.8% Christian, followed by Muslim (1.9%), Hindu, Jewish, Buddhist, and African folk religion (2%), and non-specific religious groups (15.3%) [44].

However, South Africa is an upper-middle power in international affairs. It maintains significant regional influence as a member of the Commonwealth of Nations and G20, and is ranked 114th on the Human Development Index. Since the end of apartheid, government accountability and quality of life has improved in South Africa. However, crime, poverty and inequality remain widespread, with about a quarter of the population being unemployed and living on less than US$1.25 a day as of 2008 [44]. Besides, South Africa, like many other developing countries, has quite a young population, constituting 37% of the population in 2010, totalling 19.1 million persons aged 14–35 years [1]. South African youths are plagued with challenges such as crime, unemployment, poverty, and, most importantly, unequal opportunities in education.

Similarly, the country is currently plagued with persistent droughts and water scarcity, which predominantly influence irregular labour migration. Migration within and outside countries in South Africa is driven by the pursuit of economic opportunities, political uncertainty and, increasingly, environmental hazards [1]. Thus, industrial developments such as the mining sectors in South Africa, Botswana and Zambia, and the oil wealth of Angola, have been attractive features for both skilled and unskilled labour migrants from within the region and elsewhere. In addition, Stats SA [44] reported an estimate of 2.9 million migrants that are presently residing in South Africa in mid-year 2020.

### Study design and data source

This study used data from the 2009–2021 Gauteng City-Region Observatory (GCRO) Quality of Life (QoL). The GCRO QoL is a cross-sectional sample survey conducted by the Gauteng City-Region Observatory (GCRO) in collaboration with the University of Johannesburg (UJ), the University of the Witwatersrand (Wits), the Gauteng Provincial Government (GPG), and several Gauteng municipalities (organised local government – South African Local Government Association – SALGA) in South Africa [30]. In 2009, the first Quality of Life (QoL) survey was conducted; the GCRO measures the quality of life, socioeconomic circumstances, attitudes to service delivery, psychosocial attitudes, value-base and other characteristics of the Gauteng City Region (GCR) [30–35]. Also, the GCRO QoL programme was established as a national multi-purpose household survey initiative to assist the provinces in gathering comparable national data on a wide range of initiatives about internal and international migrants in South Africa. In addition, GCRO QoL analyses key indicators for the South African provinces to generate data for national development plans, policies, and programmes, and measure progress towards SDGs and other agreements signed internationally [45, 46].

A multi-stage, stratified cluster sampling approach was used to nationally examine a household-based survey with randomly selected adults (aged 18^+^) across Gauteng as respondents [30–35]. The sampling frame for data collection was based on the 2011 Population and Housing Census (2011 PHC) of South Africa. In the first stage, guided by the definition of the 2011 PHC enumeration, the enumeration areas (EAs) were identified within the selected primary sampling units (PSUs). In each EA sample, the cataloguing of households was carried out, and a sample of households was selected in the second stage using systematic random sampling. In each household, all persons who met the inclusion criteria (i.e., adults aged 18 years and older) were eligible to participate in the survey. The GCRO conducted previous Quality of Life Surveys in 2009 (Round 1) [30], 2011 (Round 2) [31], 2013–2014 (Round 3) [32], 2015–2016 (Round 4) [33], and 2017–2018 (Round 5) [34]. Round six was conducted in 2020–2021 [35] and is the most recent round of the survey. The Round 1 survey conducted in 2009 was allocated 5,740 sampled enumeration areas (sample clusters or primary sampling units) [30], the Round 2 survey in 2011 was allocated 1,008 sampled enumeration areas [31], and Round 3 survey conducted in 2013–2014 used 16,400 sampled enumeration areas [32], while the Round 4 survey in 2015–2016 was allocated 24,889 sampled enumeration areas [33], the Round 5 survey conducted in 2017–2018 was allocated 22,220 sampled enumeration areas [34], and Round 6 survey piloted in 2020–2021 was allocated 3,075 sampled enumeration areas [35]. The sample clusters were distributed between the urban and rural strata within each municipality of the sampled enumeration areas proportionate to the size of the corresponding populations within the frame. Clusters (primary sampling units) were assigned to each area’s urban and rural strata in proportion to the number of households in the census frame for each stratum within the provinces (region). The final samples were 105,346 clusters and 73,332 households across all sampling strata [30–35].

### Study population and sample size

The study populations were made up of migrant populations stratified by internal migrants and international migrants. By conceptual clarification and operationalisation in this study, international migrants are those who move across international borders for economic or settlement purposes. By contrast, internal migrants are persons who move within a province or from one province to another in search of economic resources. However, in this study, the total number of internal migrants was 21,879 while the international migrants were 4,807, ranging from age 18 to 48^+^ years, with the males numbering 13,200 and females 13,486, totalling 26,686 for the sample size for this study [30–35]. In order to expand the range of generalisation, both migrant groups were used as the target population to increase the level of precision, hence its justification.

Thus, several studies in South Africa and elsewhere have reported that domestic internal migrants move from rural to urban areas, or from poorer areas to richer ones, in search of better job prospects. These are better documented administratively, which explains their large population size [1]. Similarly, studies have shown that international migrants are vulnerable to discrimination and exploitation, as many are poor and illiterate, and reside in slums and hazardous shelters prone to disaster and natural calamities [1, 47]. Therefore, the dearth of policies and programmes providing for the needs and settlements of migrants has led to their poor documentation and insignificant population size.

### Measures

The trained enumeration officials of the Gauteng City-Region Observatory’s Quality of Life Survey collected the data. The GCRO Quality of Life Rounds 1 to 6 (2009‒2021) [30–35] survey – Full Questionnaires were included in the field data collection instrument, which comprised questions focused on: (1) demographic details of the enumerated population (population group, gender, age, language), (2) housing (dwelling type, tenure, satisfaction with dwelling, perceived quality of housing and housing allocation), (3) household services (water, sanitation, refuse, energy sources), (4) migration, health (including disability), (5) education and employment (including employment sector), (6) community services (amenities, transport, leisure activities, safety and crime), (7) financial data (including debts, income, and social grants), (8) household assets (Telephone, Television, Computer, Radio, Music system, Satellite TV [e.g. MNET, DSTV], Internet connection, Car, Bicycle, Fridge), (9) public participation and governance, (10) perceived personal well-being, and 11) quality of life of respondents. We used data collected from questionnaires administered to randomly selected women and men living in the surveyed household [30–35].

### Outcome variable

The outcome variable is life satisfaction, and the Satisfaction with Life Scale (SWLS) was used to measure the individuals’ overall life satisfaction. The SWLS is a five-item scale developed by Diener et al. [48, 49] to evaluate people’s life. Each item is rated from 1 to 5 based on the degree of agreement with descriptions related to satisfaction with the respondent’s life: 1 = very dissatisfied; 2 = dissatisfied; 3 = neither satisfied nor dissatisfied; 4 = satisfied; 5 = very satisfied. The total score of the SWLS was calculated for each respondent suggested by Diener et al. [48]. Then, the total score of the SWLS was further measured by adopting the use of Cantril’s Self-Anchoring Ladder of Life Satisfaction scale [50] and Gallup [51], with levels numbered from ‘0’ at the bottom to ‘10’ showing respondents’ self-reported life satisfaction, as cited by Deaton [52]. The outcome variable, life satisfaction, was measured using Cantril’s Self-Anchoring Ladder of Life Satisfaction and Gallup scales. Thus, the respondents indicated their level of satisfaction with the life they believed they had and where they stood on the level of life satisfaction at the time of the survey. Following the adoption of the recommendation of Cantril’s Self-Anchoring Ladder of Life Satisfaction and Gallup scales, the respondents’ responses of the total score of the SWL were re-categorised and recoded as ‘Suffering (0–4)’ as ‘0’, ‘Struggling (5–6)’ as ‘1’, and ‘Thriving (7–10)’ as ‘2’. This re-categorised variable was kept solely for description; the ordered categorical variable (i.e., the 0 to 10 ordinal measurement) was used as Lowest (0), Middle (5) and Highest (10) in the multivariate analysis using the statistical methods of ordered logit model to calculate the life satisfaction by gender. The work on SWLS conducted by Diener et al. [48], Cantril’s Self-Anchoring Ladder of Life Satisfaction scale [50], and Gallup [51], are reliable and valid tools for measuring life satisfaction in several related studies [53, 54].

### Explanatory variables

The explanatory variables for this study were classified into individual-level, household-level and community-level factors. The individual-level factors are age, gender, education, population group, respondent’s income, wealth index, employment, occupation, marital status, access to mass media, type of dwelling, SASSA social grant, intimate partner violence, health insurance cover, and healthcare services. The household-level factors include the following: household wealth index, head of household, number of people living in the household, parity/number of children, number of people under 18 living in the household, number of people aged 60 or more living in the household, and do not have enough money to feed children. The community-level factors include residential status, migrant status, and access to media [30–35].

**Table 1:** Detailed explanations of individual-level, household-level and community-level variables categorization

Variables	Categorization
***Individual level factors***	
Age	1 = 18‒27; 2 = 28‒37; 3 = 38‒47; 4 = 48^+^years
Gender	1 = Male; 2 = Female
Education	1 = No education; 2 = Primary; 3 = Secondary/Higher
Population group	1 = Black African; 2 = Non-Black African (Coloured, White, Indian/Asian, others)
Respondent’s income	1 = No income; 2 = Low (R1-R12,800); 3 = Middle (R12,801-R25,600); 4 = High (R25,601^+^)
Employment	1 = Not working; 2 = Working
Health insurance cover	1 = No; 2 = Yes
Healthcare services	1 = Public; 2 = Private; 3 = Both public and private
Life satisfaction	0 = Suffering; 1 = Struggling; 2 = Thriving
***Household-level factors***	
Number of persons living in H/H	1 = 1; 2 = 2; 3 = 3; 4 = 4^+^persons
Number of persons under 18 in H/H	1 = 0; 2 = 1; 3 = 2; 4 = 3; 5 = 4^+^persons
Not enough to feed children	1 = No; 2 = Yes; 3 = No children in H/H
Receiving SASSA Social Grant	1 = No; 2 = Yes
***Community-level factors***	
Type of dwelling	1 = Formal; 2 = Informal
Migrant status	1 = Internal migrants; 2 = International migrants

*Source*: Authors’ compilation; H/H = Household; SA = South Africa; internal migrants are persons born in SA but not in Gauteng Province; international migrants are persons born outside South Africa.

However, these selected predictors were based on reports from prior studies and availability of the variables [17, 55]. In this study, the original categorization of some of these variables from the dataset was maintained, while others were re-categorized and recorded in order to increase precision from the analysis employed [30–35]. Most selected variables were measured simply in a binary variable with responses as ‘yes’ or ‘no’. On the other hand, others were aggregated from responses to several questions like the computation of household wealth of respondents using household characteristics, possessions and assets (e.g., internet access, number of rooms for sleeping, source of drinking water, ownership of television, radio, vehicle, and access to electricity, among others), or household wealth categorised into poorest (1), middle (2), and rich (4). Hence, detailed explanations for these variables are provided elsewhere [30–35] (See Table 1 above).

### Data preparation and analysis

**Fig. 1:**
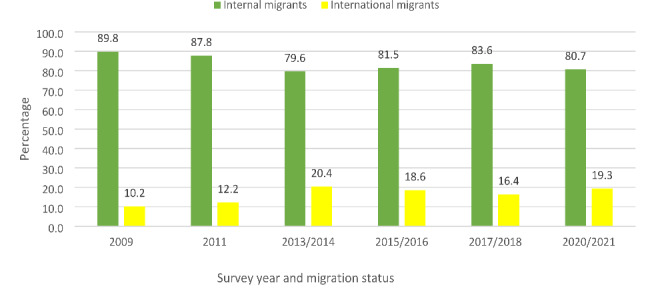
Distribution of the Trend of Persons with migrant status by survey years (2009‒2021)

**Fig. 2:**
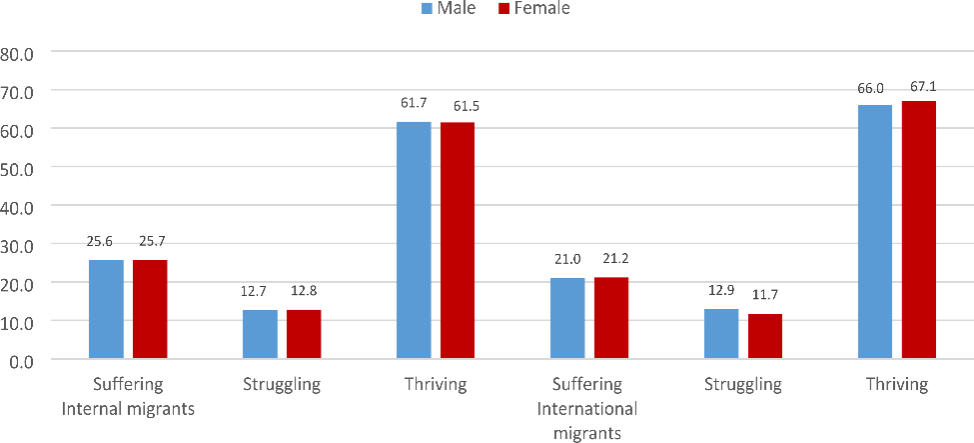
The gender distribution of life satisfaction by migrants’ status (internal and international) in South Africa (2009‒2021)

**Fig. 3:**
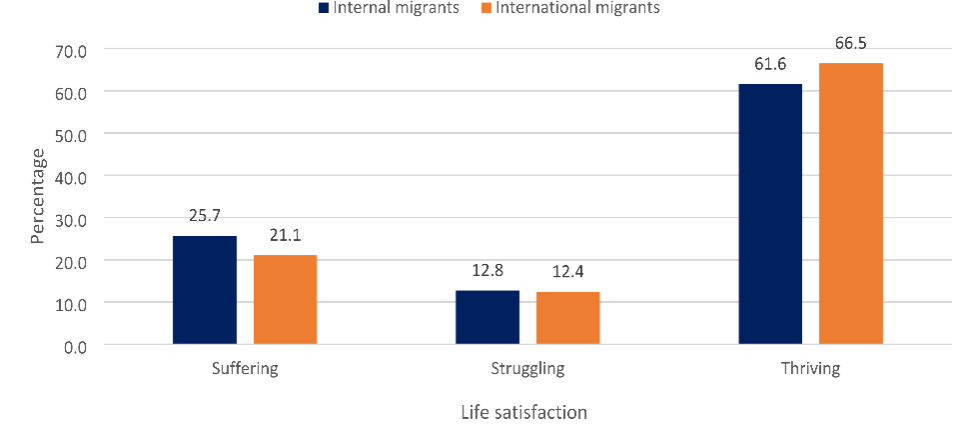
Distribution of Respondents’ Life satisfaction among internal and international migrants (2009‒2021)

The study set out to unravel the individual-level, household-level and community-level factors that determine life satisfaction among migrants in South Africa who are aged 18–48^+^ years. These measures were followed based on the study objectives to analyse the dataset. The weighting variables of each survey and the “svy command” were applied to deal with over- and under-sampling biases, and gauge the complex survey design and generalizability of the findings. The demographics of the study population stratified by internal and international migrants in South Africa (2009‒2021) (Table 2), distribution of the Trend of Persons with migrant status (internal and international) by survey years (2009‒2021) in South Africa (Fig. 1), the gender distribution of life satisfaction by migrants’ status (internal and international) in South Africa (2009‒2021) (Fig. 2) and distribution of Respondents’ Life satisfaction among internal and international migrants (2009‒2021) (Fig. 3) were calculated using the descriptive statistical analyses. This was followed by the univariate descriptive computation of the explanatory variables to show the summary statistics of the data.

After that, a cross-tabulation computation of the outcome variable across the explanatory variables (individual-level, household-level and community level) was done. The findings were presented in proportions and percentages. In addition, a cross-tabulation computation of outcome variables across the explanatory variables was done, and the findings were presented in proportions and percentages. Moreover, a Pearson’s Product Moment Correlation Coefficient (r) was performed to ascertain the degree of the relationship between life satisfaction and the determinants (individual-level, household-level and community-level) at a 5% level of significance threshold (Table 3). Finally, we performed multivariable analyses, regressing life satisfaction (as initially measured with the SWLS of Diener et al. [48], Cantril’s Self-Anchoring Ladder of Life Satisfaction scale [50] and Gallup [51]) onto the predictor variables on the full and gender-stratified samples using ordered probit and logit regression model and its commands to predict its probabilities with marginal effects for life satisfaction by gender.

The ordered probit (oprobit) model is typically used to explain the variation in an ordered categorical dependent (ordinal logistics regression) variable as a function of one or more independent variables (Table 4). Though argued in several studies to produce parameter estimates challenging to interpret, oprobit was fitted mainly for its generalisation ability to preserve the ordering of the response options in the outcome variable as a function of the explanatory variables [19, 23]. Next, we ran the margins command to produce predicted probabilities of ordered logit (ologit) models based on the cumulative probabilities of the response variable. In particular, the logit of each cumulative probability is assumed to be a linear function of the covariates, with regression coefficients constant across response categories [56, 57]. This was achieved by predicting the probabilities only for the gender-stratified models and easing the interpretation of the estimated coefficient from the ologit outputs (Table 5). Additionally, margin plots were generated for the highest level of satisfaction across the individual-level, household-level and community-level variables to further support the predicted probabilities’ interpretation. We reported only adjusted models, pegging statistical significance at p < 0.05.

### Ethics

The GCRO QoL of Round 1 to Round 6 surveys from 2009 to 2021 obtained ethical clearance from the Human Research Ethics Committee (non-medical) with protocol number: H19/11/09 from the University of the Witwatersrand, Johannesburg Research office. Verbal consent was obtained from the respondents aged 18^+^ years. All respondents were informed about the voluntary nature of participation, including confidentiality and anonymity. Respondents were also asked to sign a small hardcopy receipt confirming their participation in the study. Also, field workers had copies of a letter from the Gauteng Premier and the study ethics clearance certificate, which were provided when they thought it may be helpful in fieldwork premises.

## Results

The descriptive findings revealed that most of the respondents were internal migrants, with fluctuating lower values for international migrants observed in the GCRO QoL 2009 to 2022 survey years in South Africa (Fig. 1). Figure 2 shows the gender distributions of life satisfaction among migrants, and both internal (male – 61.7% and female – 61.5%) and international (male – 66.0% and female – 67.1%) migrants mainly reported a thriving scale of life satisfaction (Fig. 2). As shown in Fig. 3, more of the international migrants (66.5%) reported thriving on the life satisfaction scale (Fig. 3).

### Socio-demographic characteristics of respondents

The socio-demographic characteristics of this study were categorised into individual-level, household-level, and community-level factors. For the individual factors, 26,686 respondents participated in the study, comprising 21,879 internal migrants and 4,807 international migrants, and stratified by 13,200 males and 13,486 females (Table 2). A majority of both the internal (29.1%) and international (38.1%) migrants fall in the age cohorts of 28–37 years old, have secondary and higher education (internal ‒84.1% and international ‒ 87.0%), were in the Black African population group (internal ‒ 88.9% and international ‒ 79.7%), have a lower income of R1 ‒ R12,800 (internal ‒ 79.4% and international ‒78.9%), are not working (internal ‒ 62.1% and international ‒ 50.4%), with no health insurance cover (internal ‒ 86.7% and international ‒ 89.5%), and using private healthcare services (internal ‒ 59.3% and international ‒ 57.2%) (Table 2).

**Table 2:** Socio-demographic characteristics of internal and international migrants in South Africa (2009‒2021) (n = 26,685)

Factors	Migrant Status				All Migrants					
	Internal migrant		International migrant		Male sample		Female sample		Total sample	
	Freq.	%	Freq.	%	Freq.	%	Freq.	%	Freq.	%
**Independent-level factors**	**21,879**	**82,0**	**4807**	**18,0**	**13,200**	**49,5**	**13,486**	**50,5**	**26,686**	**100**
*Life satisfaction*										
{0} 1‒2: Suffering	5621	25,6	1014	21,1	3261	24,7	3374	25,0	6635	24,9
{1} 3: Struggling	2790	12,8	597	12,4	1683	12,8	1703	12,6	3386	12,7
{2} 4‒5: Thriving	13,468	61,6	3196	66,5	8256	62,5	8409	62,4	16,665	62,4
*Gender*										
Male	10,532	48,1	2668	55,5	‒	‒	‒	‒	‒	‒
Female	11,347	51,9	2139	44,5	‒	‒	‒	‒	‒	‒
*Age*										
18‒27	4522	20,7	1142	23,8	2722	20,6	2944	21,8	5666	21,2
28‒37	6368	29,1	1833	38,1	4155	31,5	4044	30,0	8199	30,7
38‒47	4812	22,0	907	18,9	2904	22,0	2815	20,9	5719	21,4
48^+^years	6177	28,2	925	19,2	3419	25,9	3683	27,3	7102	26,6
*Education*										
No education	598	2,7	175	3,6	333	2,5	440	3,3	773	2,9
Primary	2872	13,1	802	16,7	1729	13,1	1944	14,4	3673	13,8
Secondary/Higher	18,409	84,1	3830	79,7	11,138	84,4	11,102	82,3	22,240	83,3
*Population Group*										
Black African	19,459	88,9	4184	87.0	11,690	88,6	11,953	88,6	23,643	88,6
Non-Black African	2420	11,1	623	13.0	1510	11,4	1533	11,4	3043	11,4
*Respondents’ income*										
No income	1417	6.5	254	5,3	912	6,9	759	5,6	1671	6,3
Low = R1 – R12,800	17,363	79.4	3795	78,9	10,133	76,8	11,024	81,8	21,157	79,3
Middle = R12.801 – R25,600	1773	8.1	413	8,6	1168	8,8	1018	7,5	2186	8,1
High = R25,601^+^	1326	6.0	345	7,2	987	7,5	685	5,1	1672	6,3
*Employment*										
Not working	13,589	62,1	2423	50,4	6694	50,7	9318	69,1	16,012	60,0
Working	8290	37,9	2384	49,6	6506	49,3	4168	30,9	10,674	40,0
*Health insurance cover*										
No	18,974	86,7	4301	89,5	11,347	86,0	11,929	88,5	23,276	87,2
Yes	2905	13,3	506	10,5	1853	14,0	1557	11,5	3410	12,8
*Healthcare services usage*										
Public	7009	32,0	1633	34,0	4540	34,4	4103	30,4	8643	32,4
Private	12,963	59,3	2748	57,2	7364	55,8	8346	61,9	15,710	58,9
Both public and private	1907	8,7	426	8,8	1296	9,8	1037	7,7	2333	8,7
**Household-level factors**										
*No. of persons living in H/H*										
1	4489	20,5	1268	26,4	4071	30,8	1686	12,5	5757	21,6
2	4422	20,2	1188	24,7	2814	21,3	2798	20,7	5612	21,0
3	3897	17,8	962	20,0	2030	15,4	2828	21,0	4858	18,2
4+	9071	41,5	1389	28,9	4285	32,5	6174	45,8	10,459	39,2
*No. of persons under 18 in H/H*										
No under 18 in H/H	1420	6,5	422	8,8	1141	8,6	698	5,2	1839	6,9
1	9077	41,5	2342	48,7	6965	52.7	4456	33,0	11,421	42,8
2	4225	19,3	906	18,8	2060	15,6	3073	22,8	5133	19,2
3	3789	17,3	658	13,7	1698	12,9	2749	20,4	4447	16,7
4	2479	11,3	382	8.0	1050	8,0	1810	13,4	2860	10,7
5+	889	4,1	97	2,0	286	2,2	700	5,2	986	3.7
*Not enough money to feed children*										
No	13,142	60,1	2684	55,9	7198	54,5	8627	64,0	15,825	59,3
Yes	3043	13,9	447	9,3	1217	9,2	2274	16,9	3491	13,1
No children in the household	5694	26,0	1676	34,9	4785	36,3	2585	19.1	7370	27,6
*Receiving SASSA social grant*										
No	12,154	55,6	4226	87,9	9477	71,8	6902	51,2	16,379	61,4
Yes	9725	44,4	581	12,1	3723	28,2	6584	48,8	10,307	38,6
**Community-level factors**										
*Type of dwelling*										
Formal	16,371	74,8	3533	73,5	9719	73,6	10,185	75,5	19,904	74,6
Informal	5508	25,2	1273	26,5	3481	26,4	3301	24,5	6782	25,4
*Migrant status*										
Internal	‒	‒	‒	‒	10,532	79,8	11,347	84,1	21,879	82,0
International	‒	‒	‒	‒	2668	20,2	2139	15,9	4807	18,0

N = sample size; Freq. = frequency; % = percentages

By household-level factors, both internal (41.5%) and international (28.9%) migrants reported having four and more than four persons living in their households. However, 41.5% of internal migrants and 48.7% of international migrants reported having one person under 18 living in their household (Table 2). Also, most of the internal migrants (60.1%) and international migrants (55.9%) reported having enough money to feed their children, and not receiving SASSA social grants (87.9%). In addition, by community-level factors, most migrants reported having a formal dwelling place of residence (internal migrants ‒ 74.8% and international migrants ‒ 73.5%) (Table 2).

### Associated related factors (individual, household, and community) and life satisfaction of respondents with migration status

Table 3 shows that individual-level, household-level and community-level factors are associated with life satisfaction by migrant status in the Pearson Correlation Coefficient model. Findings showed that individual factors such as gender, age, education, population group, income, insurance coverage and healthcare services usage were found to be associated with life satisfaction by migration status (internal and international). Regarding internal migration status, household factors such as several persons living in households (H/H), not having enough money to feed children, and receiving SASSA social grants were associated with decreased life satisfaction among internal migrants. By community-level factors, the type of dwelling was found to have a decreased association with life satisfaction for both migrants (Table 3).

**Table 3:** Correlation between Life satisfaction and Factor-related indicators (individual, household and community) among Migrants in South Africa, 2009–2021

Factors-related indicators	Migration status					
	Internal migrants			International migrants		
	N	r	ρ-value	N	r	ρ-value
**Independent-level factors**						
Gender	21,879	2.0064	0.34	4,807	1.0092	0.51
Age	21,879	1.0828	0.00 *	4,807	2.0534	0.00 *
Education	21,879	3.0153	0.02 *	4,807	2.0624	0.00 *
Population Group	21,879	1.1335	0.00 *	4,807	1.1327	0.00 *
Income	21,879	1.1627	0.02 *	4,807	3.1525	0.00 *
Employment	21,879	0.0623	0.03 *	4,807	0.0169	0.23
Health insurance cover	21,879	1.1230	0.00 *	4,807	1.1161	0.00 *
Healthcare services usage	21,879	1.0405	0.03 *	4,807	1.0300	0.03 *
**Household-level factors**						
No. of persons living in H/H	21,879	− 0.0084	0.21	4,807	0.0113	0.42
No. of persons under 18 in H/H	21,879	− 0.0296	0.02 *	4,807	0.0074	0.60
Not enough money to feed children	21,879	− 0.0366	0.01 *	4,807	0.0236	0.09
Receiving SASSA social grant	21,879	− 0.0518	0.05 *	4,807	0.0178	0.21
**Community-level factors**						
Type of dwelling	21,879	− 0.2161	0.00*	4,807	− 0.1980	0.00 *

N = sample size; r = correlation coefficient; ρ-value =*p < 0.05

#### Multivariate ordered probit coefficients of the life satisfaction predictors by gender

Table 4 showed that the probit coefficients of the total population for the individual factors [such as age 48^+^: AOR = 2.18, 95% CI: 1.13, 3.23, secondary/higher education: AOR = 1.10, 95% CI: 0.01, 1.19, non-black African: AOR = 1.28, 95% CI: 0.21, 1.35), income: high – AOR = 1.51, 95% CI: 0.41, 1.61, and employment: AOR = 1.03, 95% CI: 0.00, 1.07]; household factors [such as number of persons living in the H/H: 2 persons – AOR = 1.05, 95% CI: 0.50, 1.10, and number of persons under 18 years in H/H: 2 persons – AOR = 2.26, 95% CI: 0.18, 2.34)]; and community factors [international migrant status (AOR = 2.12, 95% CI: 0.08, 2.16)] significantly increase the predictors, leading to higher odds of life satisfaction by gender among migrants (Table 4).

**Table 4:** Multivariate Ordered Probit (Coefficients) Model regressing Life Satisfaction on Predictor Variables Factors (individual-, household- and community-level) by Gender among Migrants in South Africa, 2009–2021

Predictor variables	Gender status				Total	
	Male		Female			
Independent-level factors	Oprobit Coefficients	(95% CI)	Oprobit Coefficients	(95% CI)	Oprobit Coefficients	(95% CI)
Life satisfaction (outcome variable)						
**Gender** Male Female	‒ ‒	‒ ‒	‒ ‒	‒ ‒	RC 0.05	RC (0.02 ‒ 0.08)
**Age (in years)** 18‒27 28‒37 38‒47 48^+^	RC 0.02 0.03 1.18	RC (0.08 ‒ 0.05) (0.05 ‒ 0.10) (0.11 ‒ 1.26) *	RC 0.00 0.00 1.17	RC (0.05 ‒ 0.06) (0.06 ‒ 0.07) (0.11 ‒ 1.20) *	RC 0.01 0.01 2.18	RC (0.05 ‒ 0.04) (0.04 ‒ 0.06) (1.13 ‒ 3.23) *
**Education** No education Primary Secondary/Higher	RC 0.11 1.08	RC (0.04 ‒ 0.25) (0.06 ‒ 1.22) *	RC 0.15 1.11	RC (0.03 ‒ 0.26) (0.00 ‒ 1.23) *	RC 0.13 1.10	RC (0.04 ‒ 0.22) (0.01 ‒ 1.19) *
**Population Group** Black African Non-Black African	RC 1.27	RC (0.17 ‒ 1.36) *	RC 1.30	RC (0.20 ‒ 1.39) *	RC 1.28	RC (0.21 ‒ 1.35) *
**Respondents’ income** No income Low (R1 ‒ R12,800) Middle (R12,801 ‒ R25,600) High (R25,601^+^)	RC 0.24 1.51 1.53	RC (0.15 ‒ 0.33) (0.39 ‒ 1.64) * (0.39 ‒ 1.67) *	RC 0.14 1.38 1.50	RC (0.05 ‒ 0.23) (0.26 ‒ 1.51) * (0.35 ‒ 1.65) *	RC 0.19 1.45 1.51	RC (0.12 ‒ 0.25) (0.36 ‒ 1.54) * (0.41 ‒ 1.61) *
**Employment** Not working Working	RC 1.05	RC (0.00 ‒ 1.10) *	RC 1.02	RC (0.03 ‒ 1.06)	RC 1.03	RC (0.00 ‒ 1.07)
**Health insurance cover** No Yes	RC 1.14	RC (0.06 ‒ 1.24) *	RC 1.13	RC (0.05 ‒ 1.21)	RC 1.13	RC (0.08 ‒ 1.19) *
**Healthcare service usage** Public Private Both public and private	RC 0.13 0.04	RC (0.18 ‒ 0.08) * (0.04 ‒ 0.13)	RC 0.14 0.01	RC (0.19 ‒ 0.09) * (0.10 ‒ 0.07)	RC 0.14 0.01	RC (0.17 ‒ 0.10) * (0.05 ‒ 0.08)
**Household-level factors**						
**No. of persons living in H/H** 1 2 3 4^+^	RC 1.03 0.06 0.07	RC (0.04 ‒ 1.09) (0.02 ‒ 0.15) (0.02 ‒ 0.16)	RC 1.06 0.00 0.04	RC (0.01 ‒ 1.13) (0.09 ‒ 0.08) (0.12 ‒ 0.05)	RC 1.05 0.03 0.01	RC (0.50 ‒ 1.10) * (0.03 ‒ 0.09) (0.05 ‒ 0.07)
**No. of persons under 18 in H/H** 0 1 2 3 4 5+	RC 1.21 1.20 0.17 0.13 0.26	RC (1.13 ‒ 0.29) * (0.09 ‒ 1.31) * (0.04 ‒ 0.29) * (0.01 ‒ 0.26) (0.07 ‒ 0.45) *	RC 1.22 1.31 0.33 0.29 0.28	RC (0.12 ‒ 1.32) * (0.19 ‒ 1.42) (0.21 ‒ 0.45) * (0.16 ‒ 0.42) (0.13 ‒ 0.42) *	RC 1.21 2.26 0.26 0.22 0.24	RC (1.15 ‒ 1.27) * (0.18 ‒ 2.34) (0.18 ‒ 0.34) * (0.13 ‒ 0.31) (0.13 ‒ 0.35) *
**Not enough money to feed children** No Yes No children in the household	RC 0.49 0.04	RC (0.57 ‒ 0.41) * (0.02 ‒ 0.10)	RC 0.45 0.11	RC (0.501 ‒ 0.40) * (0.04 ‒ 0.17) *	RC 0.46 0.07	RC (0.51 ‒ 0.42) * (0.03 ‒ 0.11) *
**Receiving SASSA social grant** No Yes	RC 0.02	RC (0.05 ‒ 0.08)	RC 0.01	RC (0.06 ‒ 0.04)	RC 0.00	RC (0.04 ‒ 0.04)
**Community-level factors**						
**Type of dwelling** Formal Informal	RC 0.40	RC (0.46 ‒ 0.35) *	RC 0.42	RC (0.46 ‒ 0.37) *	RC 0.41	RC (0.45 ‒ 0.38) *
**Migrant status** Internal International	RC 1.12	RC (0.06 ‒ 1.17) *	RC 1.12	RC (0.06 ‒ 1.18) *	RC 2.12	RC (0.08 ‒ 2.16) *

Ordered probit coefficients = probit coefficients; CI = Confidence interval; RC = Reference category; Level of significance at *p < 0.05

### Predicted probabilities of logit models of predictors of life satisfaction

Table 5 shows the logit models with the average marginal effects of the predictor variables. The dependent variables accounted for all levels of life satisfaction, and the estimations of the marginal effects created seven sets of results; however, only three points on the life satisfaction ladder – namely lower, middle, and highest – were presented in this study’s findings.

**Table 5:** Predicted Probabilities of Ordered Logit Models with Marginal Effects by Levels of Life Satisfaction by Gender among Migrants in South Africa (2009–2021)

Predictors	Male (Marginal Effects (Standard error))			Female (Marginal Effects (Standard error))		
	Lower (0)	Middle (1)	Highest (2)	Lower (0)	Middle (1)	Highest (2)
**Individual-level factors**						
*Age*						
18‒27 (RC)	0.2597	0.1307	0.6096	0.2680	0.1305	0.6015
28‒37	0.2634 *	0.1315 *	0.6050	0.2673	0.1303	0.6023 *
38‒47	0.2526	0.1291	0.6184 *	0.2668	0.1302	0.6030 *
48 + years	0.2093	0.1174	0.6734 *	0.2180	0.1181	0.6640 *
*Education*						
No education (RC)	0.2692	0.1320	0.5988	0.2902	0.1340	0.5758
Primary	0.2393	0.1251	0.6356 *	0.2459	0.1248	0.6292 *
Secondary/Higher	0.2476	0.1272	0.6252 *	0.2550 *	0.1270	0.6180 *
*Population Group*						
Black African	0.2517 *	0.1295 *	0.6188	0.2590 *	0.1290 *	0.6120
Non-Black African (RC)	0.1797	0.1078	0.7126	0.1784	0.1054	0.7162
*Respondents’ income*						
No income (RC)	0.3257	0.1449	0.5294	0.3017	0.1380	0.5602
Low = R1 – R12,800	0.2507	0.1310	0.6183	0.2586	0.1296	0.5118
Middle = R12.801 – R25,600	0.1767	0.1080	0.7153 *	0.1895	0.1099	0.7006 *
High = R25,601+	0.1714	0.1059	0.7227 *	0.1605	0.0990	0.7404 *
*Employment*						
Not working (RC)	0.2545	0.1291	0.6165	0.2561	0.1272	0.6167
Working	0.2393	0.1254	0.6353 *	0.2516	0.1262	0.6222 *
*Health insurance cover*						
No (RC)	0.2511	0.1288	0.6201	0.2575	0.1280	0.6145
Yes	0.2122	0.1183	0.6695 *	0.2211	0.1188	0.6601 *
*Healthcare services usage*						
Public (RC)	0 2262	0.1228	0.6510	0.2284	0.1212	0.6504
Private	0.2624 *	0.1318 *	0.6058	0.2674	0.1305 *	0.6021
Both public and private	0.2154	0.1197	0.6649 *	0.2368	0.1134	0.6699 *
**Household-level factors**						
No. of persons living in H/H						
1 (RC)	0.2582	0.1297	0.6121	0.2534	0.1267	0.6199
2	0.2498	0.1278	0.6224 *	0.2361	0.1225	0.6415 *
3	0.2396	0.1253	0.6351 *	0.2535 *	0.1267	0.6198
4+	0.2364	0.1245	0.6391 *	0.2640 *	0.1290 *	0.6070
*No. of persons under 18 in H/H*						
0 (RC)	0.2991	0.1375	0.5634	0.3395	0.1402	0.5203
1	0.2375	0.1248	0.6377 *	0.2685	0.1293	0.6022 *
2	0.2403	0.1255	0.6342 *	0.2427	0.1235	0.6337 *
3	0.2501	0.1278	0.6221 *	0.2364	0.1219	0.6417 *
4	0.2652	0.1314	0.6036 *	0.2487	0.1250	0.6264 *
5+	0.2257	0.1216	0.6527 *	0.2526	0.1259	0.6216 *
*Not enough money to feed children*						
No (RC)	0.2362	0.1266	0.6371	0.2344	0.1258	0.6398
Yes	0.3938 *	0.1489 *	0.4573	0.3798 *	0.1482 *	0.4721
No children in the household						
*Receiving SASSA social grant*						
No (RC)	0.2485	0.1274	0.6241	0.2533	0.1266	0.6201
Yes	0.2432	0.1261	0.6308 *	0.2561 *	0.1272 *	0.6267 *
**Community-level factors**						
*Type of dwelling*						
Formal (RC)	0.2130	0.1221	0.6649	0. 2188	0.1220	0.6593
Informal	0.3348 *	0.1474 *	0.5177	0.3479 *	0.1467 *	0.5054
*Migrant status*						
Internal (RC)	0.2543	0.1289	0.6168	0.2605	0.1282	0.6113
International	0.2208	0.1203	0.6589 *	0.2253	0.1196	0.6551 *

Coef. = Robust regression coefficient; CI = Confidence interval; RC = Reference category; Level of significance at *p < 0.05

The predicted probabilities were interpreted by comparing the categories’ probabilities to their reference category. The predicted probability for the male gender aged 38–47 years (0.6184) and 48^+^ years (0.6734) had higher odds of predicting the highest life satisfaction compared to their counterparts aged 18–27 years (0.6096 – RC). Also, the predicted probability for females aged 28–37 years (0.6023), 38–47 years (0.6030) and 48^+^ years (0.6640) predicted the highest life satisfaction compared to their counterparts aged 18–27 years (0.6015 – RC). In addition, all other explanatory factors in individual-level (primary and secondary/higher education, middle and high income, working, having health insurance, and both public and private healthcare services usage), household-level (2 to 4^+^ persons living in H/H, 1 to 5^+^ persons under 18 in H/H, and receiving SASSA social grant) and community-level (international migrant status) factors showed a significant increase of highest life satisfaction among migrants (Table 5 above).

## Discussion

This study examined determinants of life satisfaction among migrants in South Africa aged 18 to 48^+^ years. In the full sample multivariable model, independent-level factors (gender, age, education, population group, respondents’ income, employment, health insurance coverage and healthcare services usage), household-level factors (number of persons living in H/H, number of persons under 18 in H/H, not enough to feed children, and receiving SASSA social grant), and community factors (type of dwelling and migrant status) were significantly associated with life satisfaction. The same relationships existed in the gender-stratified samples. However, there were some slight variations across genders, which this study will focus on in this section.

Within the age groups of migrants, those aged 28 ‒ 37, 38 ‒ 47, and 48^+^ years had a reduced probability of being satisfied with life compared with those aged 18 ‒ 27 years. We found that the findings were similar for both male and female migrants. However, internal migrant women reported lower levels of life satisfaction, while international migrant men reported lower levels of life satisfaction. Also, older migrant males and females aged 48^+^ years, irrespective of their migrant status, showed thriving life satisfaction more than younger migrant counterparts (18 ‒ 27 years). This finding on the variations of life satisfaction among migrants across age patterns revealed that happiness declines from late adolescence and rises in mid-life [58, 59]. However, the transition from adolescence to early adulthood among migrant groups is a vulnerable period. It is more prone to risks in which young people take their first tentative steps towards gaining their freedom via migration. This phase, 18 ‒ 27 years, is often associated with significant life changes and experiences as well as responsibilities, as the individual works toward his or her goals, including getting a higher education, liberation, getting married, getting a paid job, and migrating from one environment to another [60, 61]. Therefore, actualising these goals puts a burden on and induces anxiety in young migrants, and severely impairs their well-being and life satisfaction [62, 63].

The picture of migration in South Africa and globally is vastly more complicated than seen at first glance. Improved technology, rapid and accessible new forms of communication, increasing social inequality, a changing climate, a growing world economy, and greater ease of movement across the globe promise even greater complexity in the future. As these factors have accelerated and reached more corners of the globe, migration in South Africa and globally has changed [64, 65]. Migrants have begun working in industries and communities that, for many years, did not rely on migrant workers. The new migrants often do not have experience in the field where they have found work; the employers in these communities are often not equipped to communicate the health and safety risks associated with the work they offer in a linguistically and culturally appropriate way [24, 29]. The changes and increases in migration patterns, the arrival of migrants into new communities, and participation in new and often dangerous forms of employment strongly affect mobile populations’ health, health risks, and health management. In responding to life stressors, some migrants may engage in risky health behaviours such as substance use [62, 66]. Engaging in social vices such as frequent use of substances such as smoking cigarettes, marijuana, or drinking alcohol is more common among young migrants than migrants who are older [66, 67]. For older migrant females aged 48 + years, fertility declines, and the onset of menopausal symptoms, such as hot flashes, depression, and insomnia, may explain their decrease in life satisfaction [22, 68].

Also, population migration plays a critical role in disease spread by initiating acute disease outbreaks, changing the prevalence of infectious diseases at a given location, and changing the face of chronic disease resulting from a previous infection. Importantly, immigrants have ongoing links with populations in their countries of origin that may provide a channel through which infectious diseases can be introduced to new areas. Migrants are a heterogeneous group of persons, characterised by their specific language and cultural identities [69, 70], with specific health needs. Although the evidence-based information remains limited, they are at an increased risk of, and disproportionately affected by, certain communicable diseases, including tuberculosis (TB), HIV, hepatitis B and hepatitis C [70–75]. Other studies have also cited that migrants face non-communicable diseases due to their exposure to stress and hardships during their movement from one place to another [72–77]. Evidence suggests that NCDs rates differ between migrants and the host populations in host countries of residence. Several studies from cross-national comparative research have indicated that migration-related lifestyle changes associated with the lifestyle of the host population in the country of settlement may influence NCD risk among migrants in a significant way [78–83]. This suggests the need for more work to disentangle the fundamental migration-related lifestyle changes and contextual factors that may drive the differential risk of NCDs among migrants, to assist in the prevention and clinical management of NCDs in these populations.

Individual-level factors such as age (48^+^ years old), education (secondary/higher), population group (non-black African), income, health insurance cover, and health service usage (private) were related to thriving life satisfaction for both males and females by migrant status. This finding agrees with that of Whitley et al. [84] and Xiong et al. [85], who reported higher levels of life satisfaction among migrants with these individual-level factors. For instance, Maslow’s hierarchy of needs illustrates that after satisfying basic needs such as food, water, shelter, and clothing, the following higher needs of humans are the individual level factors, which include higher education and income, among others. For instance, higher education serves as a springboard for better career opportunities and reduces the risk of unemployment [86]. Moreover, better job opportunities and higher incomes are indirect channels through which higher education increases life satisfaction [87]. Hence, the gratification of this need comes with higher levels of life satisfaction, as one has a better chance of a higher income with a well-paid job due to higher educational attainment [15, 55]. However, suffering or struggling life satisfaction levels often come with anger, frustration and unhappiness, usually associated with unemployment and lower wages [15, 84] among migrants, irrespective of their visa status. This finding is consistent with previous research indicating that higher education is significantly related to the degree to which both men and women are satisfied with their lives [15, 87].

We also find that household-level factors such as the number of persons under 18 years old in households, not having enough money to feed children, and not receiving SASSA social grants, and community-level factors such as an informal type of dwelling, were found among migrant males and females who reported suffering and struggling levels of life satisfaction. This supports the findings of Agyekum [16], who found that the household-level and community-level factors mentioned above are negatively associated with a lack of contentment and lower life satisfaction in the South African context; the findings of the studies of Ebrahim et al. [3] and Meyer et al. [4] supported these study findings. The household- and community-level factors are significant critical social determinants humans want to achieve in their daily lives and dreams. When they cannot achieve these, they think less of belonging with people and within society. This brings people to the level of losing their lives to low satisfaction. Thus, many people like to set expectations for themselves, and they set a limit on their abilities and how they can achieve these [59]. Notably, the human brain can sometimes heed those beliefs, which brings constant frustrations when one feels that he/she has come up short of his/her expectations.

That is why many individuals who have many expectations to be fulfilled and cannot meet these expectations are faced with the negative feeling of lack of life satisfaction [88]. Thus, life satisfaction is one of the most critical factors that can affect migrants’ mental health and social relations, as the concepts of subjective well-being and life satisfaction have been stated by many scientists and researchers as the main goals of life and expectation of migrants, irrespective of their visa status [88]. Significantly, many factors may influence the subjective well-being and life satisfaction of internal and international migrants differently. However, these factors, such as socio-demographic and psychosocial, are often neglected when addressing the needs of migrants in developed and developing countries, such as South Africa [89–91]. Thus, individuals such as migrants tend to gain life satisfaction as they get older after building a lot of high expectations that spread across their younger-middle age to older adulthood.

The study findings also showed the importance of gender differences among migrants and their level of life satisfaction. Most internal migrant women reported more suffering and struggling levels of life satisfaction, while international male migrants reported more suffering and struggling levels of life satisfaction. Studies have indicated that gender differences in life satisfaction have been found to be significant, but however in small proportion [58, 59]. Women have reported higher levels of life satisfaction than men across all educational status, employment groups, and income levels. Thus, the direction of gender differences in life satisfaction was inconsistent across age and migrant status. Non-migrant women have choices on how to lead their lives in terms of political, economic, and social factors, which may signal a net increase of liberty and autonomy among them and, at the same time, may influence migrant women in terms of gaining empowerment to achieve their expectations and future dreams [60, 61].

Globally, women have been cited in several studies to have higher levels of life satisfaction than men, yet at the same time, they also reported more daily stress. This finding is in contrast to this study’s findings as it does not hold in countries where gender rights are compromised, but across some countries on average, the gap between male and female well-being is widening regarding educational status, wealth index, age (older women) and residing in rural areas [61, 63]. At the same time, there seems to be a modest gender difference in life satisfaction, which does not hold when women’s rights are compromised. Personal experiences influence gender identity throughout the socialisation process, the people with whom migrants relate, and their own choices, as this will ultimately bring an understanding of the gender roles and traits for males and women that are dynamic in different ways. Unequal societies are less organised, and this pushes higher rates of anti-social behaviour and violence [92, 93].

However, nations with greater gender equality are more connected, and women from such countries are better-off, with improved welfare, and have better well-being. In order to promote gender equality among migrants in South Africa, increasing women’s representation and decision-making helps in redistributing care work. Productive resources will build progress towards a gender-equal and sustainable future along with life satisfaction [63, 94]. Gender equality demands that males and females have equal freedom to choose the life they want, unhindered by gender stereotypes, roles, and prejudices, and that migrant rights, responsibilities and opportunities should not be determined by their gender, whether male or female. Gender awareness is essential because no one can ever completely step outside the social and cultural processes that partly shape our identities, values and perceptions. However, migrants have still managed to develop ways of reflecting and examining themselves, leading towards better life satisfaction despite all odds in their host countries or regions [57, 95]. Gender equality prevents violence against migrant females, which is essential for life satisfaction, and societies that value migrant women and men equally as citizens of the host countries are promoting gender equality as a human right for the host country.

Recent studies on the life satisfaction of migrants explore gender differences but yield irregular patterns which do not consider gendered sources of satisfaction. While female migrants’ rights have advanced in debates in high-income countries, there are still many poor migrant women worldwide whose lives and well-being remain compromised for the foreseeable future [67, 93]; furthermore, as the trajectory of these nations that have already improved equity in gender rights shows, the process is far from simple and does not end with legal changes alone but with sensitisation and policies that protect migrants from such crises. The rational assumption is that if the rights of migrants, irrespective of their gender, are improved, their life satisfaction levels will increase.

Our study findings further revealed that household-level factors significantly determine life satisfaction among internal and international migrants. According to the findings, males with more than four persons living in the household, not having enough to feed their children and not receiving any SASSA social grant had lower levels of satisfaction compared to those who do not have any of the indicators, while male migrants having four persons under 18 years old living with them are likely to experience middle life satisfaction. We argue that both internal and international migrants face psychological needs and are more concerned with financial burdens, leading to decreasing odds of attaining thriving life satisfaction in the long run [15, 67]. The expectations of male respondents towards achieving thriving life satisfaction differ from those of females. In addition, females with household factors such as having three or more persons living with them, having enough money to feed children, and receiving a SASSA social grant reported lower odds of experiencing middle or thriving life satisfaction.

However, females with several persons under 18 living with them in a household experience increased odds of middle or thriving life satisfaction. This study’s findings on gender differences towards household-level factors explain the relevance of self-identification in traditional gender roles of masculinity and femininity in women’s and men’s levels of life satisfaction [96, 97]. Nevertheless, the well-being of migrants should not be measured using objective well-being alone, as gender equality will be seen as one-sided, which implies that the evaluations of the living conditions of migrants, irrespective of their status, are usually ignored. Several studies have found that in both genders, the most important predictors of life satisfaction were self-esteem and social support. Both masculinity and femininity were associated with life satisfaction in males and females [98, 99]. Besides predicting life satisfaction, femininity interacted with social support in women and masculinity with self-esteem in males. It was found that the association between femininity and life satisfaction only occurred in migrant females with high social support. Self-esteem was associated with life satisfaction only in men with low masculinity.

Finally, community factors, such as type of dwelling (formal and informal) were associated with both male and female migrants who are residing in an informal type of dwelling being less likely to experience thriving life satisfaction [100, 101], while both male and female migrants of international status were associated with the predicted outcome that they are more likely to experience thriving life satisfaction. Although reasons for these decreased odds of aspiring to have thriving life satisfaction among both migrants may not be fully known to the authors, a combination of factors, including religiosity and high levels of connectedness, may be contributing to the lower odds of life satisfaction [12, 14]. The increased odds of international migrants experiencing thriving life satisfaction is predominantly expected, as they have the purpose of moving from their own country to another country.

Also, as international migrants usually change their place of residence from one country to another, they tend to have higher expectations in building a thriving life satisfaction around them and their households. Studies have supported this finding and explained that international migrants usually have higher expectations and are usually seen working towards achieving their dreams of a thriving life satisfaction [102, 103]. This exciting finding suggests that migrant status significantly predicts the highest life satisfaction of migrant males and females. Despite not knowing the exact reason for this change, we believe that this may be a reflection of the reality of international migrants in South Africa, who migrated so long ago to their host country that they no longer view migrant status as a factor in determining their own happiness and life satisfaction, and have subsequently decided to stay for a long time in South Africa in trying to meet up with their expectations of achieving higher life satisfaction for themselves and for their families as well.

Regarding the bottom-up theoretical perspective, satisfaction with domains are consistent with one’s values, which is demonstrated to be more significant with the overall satisfaction of migrant populations in South Africa. Individuals who place a high value on success and those who place a high value on relationships lay more emphasis on job and family satisfaction in the assessment scale of life satisfaction. However, the influences of demographic factors and psychosocial variables should be taken into consideration, especially when these factors do not predict life satisfaction [40, 104]. Similarly, a substantial amount of research has been conducted using a variety of methodological approaches to determine what influences life satisfaction of migrants in South Africa. The bottom-up theory considers overall satisfaction a function of situational or dispositional influences or characteristics that may influence migrants’ behaviours towards achieving high life satisfaction. Thus, the situational influences are the external factors that have an influence on an individual’s behaviour, while dispositional factors refer to the internal characteristics of an individual that may influence their behaviour. Consistent with other studies, we found that the bottom-up theory of life satisfaction are supported by studies in Germany [53], China [39], United Kingdom [42], and Chile [37] by demonstrating the demographics, levels of life satisfaction, and other psychosocial factors can explain a significant portion of variances in overall levels of life satisfaction. We propose that future psycho-demographic studies in relation to life satisfaction should consider an integrated account of life satisfaction rather than a lone bottom-up perspective.

### Strengths and limitations

One strength of this paper is its ability to stratify established relationships along gender lines, generating richer information about the determinants of life satisfaction among internal or international migrants, stratified by gender, in South Africa. Another strength is the study’s use of a nationally representative dataset (GCRO QoL 2009‒2021), which facilitates generalisation and enhances reliability by reducing the effects of potential errors induced by self-reporting. Nevertheless, these findings should be interpreted cautiously owing to a few limitations. First, using a cross-sectional study limits the ability to assess the trends and establish causation between the various factors and life satisfaction. Therefore, it is recommended that the associated factors (individual-level, household-level and community-level) explored in this study should be studied longitudinally.

Also, future studies conducted on migration studies in South Africa should attempt to use other robust analyses such as multilevel modelling and testing interaction effects (e.g., age-gender interaction). Secondly, we may have made some errors of omission in our model after some variables were not found in individuals’ GCRO QoL 2009‒2021 (such as marital status, religion, etc.). However, this omission occurred since data on these variables were not collected from the participants aged 18 ‒ 49^+^ years old. Therefore, the GCRO should endeavour to include these missing variables for individuals aged 18 ‒ 49^+^ years old in future datasets. Finally, we would also like to mention that our results only extend previous literature on life satisfaction among migrants in South Africa.

## Conclusion

This study presents findings suggesting that factors at the individual-level (such as age, education, population group, income, employment, health insurance cover, and health services usage), household-level (persons living in a household, persons under 18 years old living in a household, not having enough money to feed children, and not receiving a SASSA social grant), and community-level (informal type of dwelling and international migrant status) are determinants of life satisfaction or thriving among migrants in South Africans. It is also reported that this pattern of relationships varied slightly between male and female migrants. These findings collectively provide helpful information for policymakers, practitioners, and researchers, for instance, in the formation of policies towards providing equitable and equivalent support across genders, taking into consideration the intricate associations between determinants and life satisfaction as established in this study.

Evidence from this study also calls on the government and relevant stakeholders of South Africa to begin tracking the life satisfaction of migrants, as in recent times, the inclusion of self-reported well-being and life satisfaction in governmental policies for tracking objective social and economic progress has been advocated. Owing to the findings of this study, various countries and international migration organisations have taken necessary steps to make life satisfaction central to development policies. For instance, the Sustainable Development Goals are the blueprint for achieving a better and more sustainable future for all, including migrants. The three main focus of the SDGs are: Goal 1: No Poverty. To end poverty in all its forms everywhere. Goal 2: Zero Hunger. To end hunger, achieve food security and improved nutrition and promote sustainable agriculture. Goal 3: Good Health and Well-being. To ensure healthy lives and promote well-being for all ages. Therefore, our study findings have provided a step towards this realisation among migrants in South Africa.

### Electronic supplementary material

Below is the link to the electronic supplementary material.

Supplementary Material 1

Supplementary Material 2

Supplementary Material 3

Supplementary Material 4

Supplementary Material 5

Supplementary Material 6

Supplementary Material 7

Supplementary Material 8

Supplementary Material 9

## Data Availability

The data that support this analysis are available at the DataFirst service at UCT and can be accessed from https://www.datafirst.uct.ac.za/dataportal/index.php/collections/GCRO subject to permission or request from the GCRO.

## Abbreviations

TB: Tuberculosis
HIV: Human Immunodeficiency Virus
NCDs: Non-communicable diseases
GCRO: The Gauteng City Observatory
SDGs: Sustainable Development Goals
QoL: Quality of Life
WHO: World Health Organization

## References

[1] Statistics South Africa (Stat SA). Draft National Labour Migration Policy for South Africa. Statistics South Africa., 2022, 115 p. Accessed on the 18th March 2022 from https://www.labour.gov.za/DocumentCenter/Publications/Public%20Employment%20services/National%20Labour%20Migration%20Policy%202021%202.pdf.

[2] Stern R, Puoane T, Tsolekile L (2010). An exploration into the determinants of non-communicable diseases among rural-to-urban migrants in peri-urban South Africa. Prev Chronic Dis.

[3] Ebrahim A, Botha F, Snowball J. Determinants of life satisfaction among race groups in South Africa. Volume 30. Development Southern Africa; 2013. 2.

[4] Meyer DF, Dunga SH (2014). The determinants of life satisfaction in a Low-Income, Poor Community in South Africa. Mediterranean J Social Sci.

[5] Statistics South, Africa. 2021. Annual Report 2020/2021 (Book 1) / Statistics South Africa. Pretoria: Statistics South Africa, 2021, 242 p. Published by Statistics South Africa, Private Bag X44, Pretoria 0001. Accessed on the 14th January 2023 from https://www.statssa.gov.za/publications/AnnualReport/Annual%20Report%20Book%201202021_Final_Web.pdf.

[6] Statistics South Africa (Stat SA). Census 2011 Statistical release – Mid-year population estimates 2011. Statistics South Africa (Stat SA), 2011/P0301.4. Published by Statistics South Africa, Private Bag X44, Pretoria 0001. Accessed on the 10th of. December 2022 from https://www.statssa.gov.za/publications/P03014/P030142011.pdf.

[7] Paparusso A (2019). Studying immigrant integration through self-reported life satisfaction in the country of Residence. Appl Res Qual Life.

[8] Hajak VL, Sardana S, Verdeli H, Grimm SA (2021). Systematic review of factors affecting Mental Health and Well-Being of Asylum Seekers and Refugees in Germany. Front Psychiatry.

[9] Kristiansen M, Razum O, Tezcan-Güntekin H, Krasnik A (2016). Aging and health among Migrants in a european perspective. Public Health Rev.

[10] Schapendonk J, van Liempt I, Schwarz I, Steel G (2020). Re-routing Migration Geographies: Migrants, Trajectories and mobility regimes. Geoforum.

[11] Switek M. Internal migration and life satisfaction: Wellbeing effects of moving as a young adult. Institute for the Study of Labor (IZA), Bonn. IZA Discussion Papers, 2012, No. 7016.

[12] Vito ED, Waure CD, Specchia ML, Ricciardi W. Public health aspects of migrant health: a review of the evidence on health status for undocumented migrants in the European Region. Volume 42. World Health Organization. Regional Office for Europe. Health Evidence Network synthesis report; 2012. p. 36. 27536764

[13] Jackson Y, Courvoisier DS, Duvoisin A, Ferro-Luzzi G, Bodenmann P, Chauvin P, Guessous I, Wolff H, Cullati S, Burton-Jeangros C (2019). Impact of legal status change on undocumented migrants’ health and well-being (parchemins): protocol of a 4-year, prospective, mixed methods study. BMJ Open.

[14] White A, Dito BB, Veale A, Mazzucato V (2019). Transnational migration, health and wellbeing: nigerian parents in Ireland and the Netherlands. Comp Migration Stud.

[15] Ciorbagiu I, Stoica A, Mihaila M (2020). Life satisfaction and migration - what relationship?. J Social Economic Stat.

[16] Agyekum BA (2020). Logistic regression analysis of life satisfaction amongst African Immigrants in Hamilton, Canada. Soc Without Borders.

[17] Owusu AK, Dey NEY, Adade AE, Agbadi P. Determinants of life satisfaction among Ghanaians aged 15 to 49 years: a further analysis of the 2017/2018 multiple cluster Indicator Survey. PLoS ONE, 2022, 17 (1), e0261164. 10.1371/journal.pone.0261164 PMC8782464 35061700

[18] Botha F, Booysen F (2013). The relationship between marital status and life satisfaction among south african adults. Acta Academica.

[19] Ngoo YT, Tan EC, Tey NP (2020). Determinants of life satisfaction in Asia: a Quantile Regression Approach. J Happiness Stud.

[20] Collinson MA, White MJ, Ginsburg C, Gómez-Olivé FX, Kahn K, Tollman SM (2016). Youth migration, livelihood prospects and demographic dividend: a comparison of the census 2011 and Agincourt health and demographic surveillance system in the rural north-east of South Africa. Afr Popul Stud.

[21] Angelini V, Casi L, Corazzini L (2015). Life satisfaction of immigrants: does cultural assimilation matter?. J Popul Econ.

[22] Ginsburg C, Collinson MA, Iturralde D, van Tonder L, Gómez-Olivé FX, Kahn K, Tollman S (2016). Migration and settlement change in South Africa: triangulating census 2011 with longitudinal data from the Agincourt health and demographic surveillance system in the rural north-east. South Afr J Demogr.

[23] Ginsburg C, Collinson MA, Gómez-Olivé FX, Gross M, Harawa1 S, Lurie MN, Mukondwa K, Pheiffer CF, Tollman S, Wang R, White MJ. Internal migration and health in South Africa: determinants of healthcare utilisation in a young adult cohort. BMC Public Health. 2021;21:554. 10.1186/s12889-021-10590-6 PMC7981972 33743663

[24] Refaeli T, Weiss-Dagan S, Levy D, Itzhaky H (2022). We are Young, we run Free: Predicting factors of life satisfaction among young backpackers. Int J Environ Res Public Health.

[25] Switek M (2016). Internal migration and life satisfaction: well-being paths of young adult migrants. Soc Indic Res.

[26] Tao L, Wong FKW, Hui ECM (2014). Residential satisfaction of migrant workers in China: a case study of Shenzhen. Habitat Int.

[27] Ekholuenetale M, Benebo FO, Idebolo AF. Individual-, household-, and community-level factors associated with eight or more antenatal care contacts in Nigeria: evidence from demographic and Health Survey. PLoS ONE, 2020, 15 (9), e0239855. 10.1371/journal.pone.0239855 PMC7518602 32976494

[28] Wen M, Wang G (2009). Demographic, psychological, and social environmental factors of loneliness and satisfaction among rural-to-urban migrants in Shanghai, China. Int J Comp Sociol.

[29] Reardon C, George G. An examination of the factors fueling migration amongst Community Service practitioners. Afr J Prm Health Care Fam Med., 2014, #625, 6 (1), 1–9. 10.4102/phcfm.v6i1.625 PMC4502841 26245415

[30] Gauteng City-Region Observatory (GCRO). (2009). GCRO Quality of Life Survey I (2009). Gauteng City-Region Observatory, October 2009. Accessed on January 14th 2023, from https://cdn.gcro.ac.za/media/documents/GCRO_2009_QoL_survey_Life_in_the_city-region.pdf.

[31] Gauteng City-Region Observatory (GCRO), Life Survey II. (2011). GCRO Quality of (2011). Gauteng City-Region Observatory, October 2011. Accessed on January 14th, 2023, from https://gcro.ac.za/research/project/detail/quality-of-life-survey-ii-2011/.

[32] Gauteng City-Region Observatory (GCRO). (2013). GCRO Quality of Life Survey III (2013). Gauteng City-Region Observatory, October 2013. Accessed on January 14th, 2023, from https://gcro.ac.za/research/project/detail/quality-of-life-survey-iii-2013/.

[33] (2015/16). Quality of Life Gauteng City-Region Observatory (GCRO), Survey IV. (2015). GCRO Quality of Life Survey IV (2015). Gauteng City-Region Observatory, October 2015. Accessed on January 14th, 2023, from https://gcro.ac.za/research/project/detail/quality-of-life-survey-iv-2015/.

[34] (2017/18). GCRO Quality of Life Gauteng City-Region Observatory (GCRO), Survey V. (2017/2018). Gauteng City-Region Observatory, October 2018. Accessed on January 14th, 2023, from https://gcro.ac.za/research/project/detail/quality-of-life-survey-v-201718/.

[35] Gauteng City-Region Observatory (GCRO). (2020/21). GCRO Quality of Life Survey VI (2020/21). Gauteng City-Region Observatory, October 2021. Accessed on January 14th, 2023, from https://gcro.ac.za/research/project/detail/quality-life-survey-vi-202021/.

[36] Diener E (1984). Subjective well-being. Psychol Bull.

[37] Loewe N, Bagherzadeh M, Araya-Castillo L, Thieme C, Batista-Foguet JM (2014). Life domain satisfactions as predictors of overall life satisfaction among workers: evidence from Chile. Soc Indic Res.

[38] Montag UK, Panksepp GA (2017). Primary emotional systems and personality: an evolutionary perspective. Front Psychol.

[39] Gan S-W, Ong LS, Lee CH, Lin YS (2020). Perceived social support and life satisfaction of malaysian chinese young adults: the mediating effect of loneliness. J Genet Psychol.

[40] Van Aardt CJ, De Clercq B, Meiring J (2019). The stochastic determinants of happiness in South Africa: a micro-economic modelling approach. J Economic Financial Sci.

[41] Urquijo I, Extremera N, Azanza G (2019). The contribution of emotional intelligence to career success: beyond personality traits. J Environ Res Public Health.

[42] Zhou M, Lin W (2016). Adaptability and life satisfaction: the moderating role of Social Support. Front Psychol.

[43] Statistics South Africa (Stat SA). Census 2011: Migration Dynamics in South Africa. Statistics South Africa (Stat SA), 2015, Report No. 03-01-79, 215 p. Published by Statistics South Africa, Private Bag X44, Pretoria 0001. Accessed on the 10th of December 2022 from https://www.statssa.gov.za/publications/Report-03-01-79/Report-03-01-792011.pdf.

[44] Statistics South Africa (Stat SA). Statistics South Africa releases results of 2020 General Household Survey – 2020 mid-year population estimates. Statistics South Africa (Stat SA), 2020. Published by Statistics South Africa, Private Bag X44, Pretoria 0001. Accessed on the 10th of. December 2022 from https://www.gov.za/speeches/statistics-south-africa-release-results-2020-general-household-survey-2-dec%C2%A0-1-dec-2021.

[45] Mercandalli S, Losch B, Belebema MN, Bélières J-F, Bourgeois R, Dinbabo MF, Fréguin-Gresh S, Mensah C, Nshimbi CC Rural migration in sub-Saharan Africa, editors. Patterns, drivers and relation to structural transformation. Rome, FAO and CIRAD, 2019.

[46] Akanle O, Kayode D, Abolade I (2022). Sustainable development goals (SDGs) and remittances in Africa. Cogent Social Sciences.

[47] Human Sciences Research Council (HSRC). Migration scholars release statement on international migration situation in South Africa. The HSRC Research Data Service, 2022. Accessed on the 10th of. December 2022 from https://hsrc.ac.za/press-releases/dces/migration-scholars-release-statement-on-international-migration-situation-in-south-africa/.

[48] Diener E, Emmons RA, Larsen RJ, Griffin S (1985). The satisfaction with Life Scale. J Pers Assess.

[49] Diener E, Pressman SD, Hunter J, Delgadillo-Chase D (2017). If, why, and when Subjective Well-Being Influences Health, and future needed research.

[50] Cantril H (1965). The pattern of human concerns.

[51] Gallup. World Poll Methodology. Technical Report, 2009. Washington, DC.

[52] Deaton A, Income (2008). Health, and well-being around the World: evidence from the Gallup World Poll. J Economic Perspect.

[53] Milovanska-Farrington S, Farrington S, Happiness (2022). Domains of life satisfaction, perceptions, and valuation differences across genders. Acta Psychol.

[54] Prati A, Senik C (2022). Feeling good is feeling better. Psychol Sci.

[55] Joshanloo M. Gender differences in the predictors of life satisfaction across 150 nations. Pers Indiv Differ, 2018, 135.

[56] López-Ortega M, Torres-Castro S, Rosas-Carrasco O (2016). Psychometric properties of the satisfaction with Life Scale (SWLS): secondary analysis of the Mexican Health and Aging Study. Health Qual Life Outcomes.

[57] Espejo B, Martín-Carbonell M, Checa I, Paternina Y, Fernández-Daza M, Higuita JD, Albarracín A (2022). Cerquera, A. Psychometric Properties of the Diener satisfaction with Life Scale with five response options Applied to the colombian Population. Front Public Health.

[58] Al-Attiyah A, Nasser R (2016). Gender and age differences in life satisfaction within a sex-segregated society: sampling youth in Qatar. Int J Adolescence Youth.

[59] Calasanti T, Carr D, Homan P, Coan V (2021). Gender disparities in life satisfaction after Retirement: the Roles of Leisure, Family, and finances. Gerontologist.

[60] Montgomery M. Reversing the Gender Gap in Happiness. CESR-Schaeffer Working Paper No. 003. Journal of Economic Behavior and Organization, 2022; 96, 65–78.

[61] Becchetti L, Conzo G (2022). The gender life Satisfaction/Depression Paradox. Soc Indic Res.

[62] Pronk NP, Kottke TE, Lowry M, Katz AS, Gallagher JM, Knudson SM, Rauri SJ, Tillema JO (2016). Concordance between life satisfaction and six elements of well-being among respondents to a health assessment survey, HealthPartners employees, Minnesota, 2011. Preventing Chronic Disease - Public Health Research Practice and Policy.

[63] Pérez-Urdiales I, Goicolea I, Sebastián MS, Irazusta A, Linander I (2019). Sub-saharan african immigrant women’s experiences of (lack of) access to appropriate healthcare in the public health system in the Basque Country, Spain. Int J Equity Health.

[64] Dumludag D (2015). Consumption and life satisfaction at different levels of economic development. Int Rev Econ.

[65] Hsu C–Y, Chang S-S, Yip P (2017). Individual-, household- and neighbourhood-level characteristics associated with life satisfaction: a multilevel analysis of a population-based sample from Hong Kong. Urban Stud.

[66] Manyisa ZM, van Aswegen EJ (2017). Factors affecting working conditions in public hospitals: a literature review. Int J Afr Nurs Sci.

[67] Majee W, Dinbabo MF, Ile I, Belebema MA (2019). Immigrant and Refugee families’ perceptions on informational support and health status: a comparison of african immigrants living in South Africa and the United States. Afr Hum Mobil Rev.

[68] Wentzel M, Tlabela K, Kok P, Gelderblom D, Oucho JO, Van Zyl J (2006). Historical background to south african migration. Migration in South and southern Africa: dynamics and determinants.

[69] Kunst AE, Stronks K, Agyemang CI, Rechel B (2011). Non-communicable diseases. Migration and health in the European Union.

[70] Rahman A, Biswas J, Banik PC (2022). Non-communicable diseases risk factors among the forcefully displaced Rohingya population in Bangladesh. PLOS Glob Public Health.

[71] Tan ST, Amanda Low PT, Howard N, Yi H (2021). Social capital in the prevention and management of non-communicable diseases among migrants and refugees: a systematic review and meta-ethnography. BMJ Global Health.

[72] Akokuwebe ME, Odimegwu C (2019). Socioeconomic determinants of knowledge of kidney disease among residents in nigerian Communities in Lagos State, Nigeria. Oman Med J.

[73] Akokuwebe ME, Odimegwu C, Omololu F, Prevalence (2020). Risk-inducing lifestyle, and perceived susceptibility to kidney diseases by gender among nigerian residents in South Western Nigeria. Afr Health Sci.

[74] Akokuwebe ME, Idemudia ES, Lekulo AM, Motlogeloa OW. Determinants and levels of cervical cancer screening uptake among women of reproductive age in South Africa: evidence from South Africa demographic and Health Survey data, 2016. BMC Public Health, 2021, 21, 2013. 10.1186/s12889-021-12020-z PMC8571865 34740352

[75] Akokuwebe ME, Idemudia ES (2021). Prevalence and socio-demographic Correlates of Body Weight Categories among South African Women of Reproductive Age: a cross-sectional study. Front Public Health.

[76] Rechel B, Mladovsky P, Ingleby D, Mackenbach JP, McKee M (2013). Migration and health in an increasingly diverse Europe. Lancet.

[77] Sohail QZ, Chu A, Rezai MR, Donovan LR, Ko DT (2015). Tu. J.V. The risk of Ischemic Heart Disease and Stroke among immigrant populations: a systematic review. Can J Cardiol.

[78] Agyemang C, Addo J, Bhopal R, de Graft Aikins A, Stronks K (2009). Cardiovascular disease, diabetes and established risk factors among populations of sub-saharan african descent in Europe: a literature review. Global Health.

[79] Byberg S, Agyemang C, Zwisler AD, Krasnik A, Norredam M (2016). Cardiovascular disease incidence and survival: are migrants always worse off?. Eur J Epidemiol.

[80] Akokuwebe ME, Idemudia ES. Knowledge and Risk Perceptions of Chronic Kidney Disease Risk Factors among Women of Childbearing Age in Lagos State, Nigeria: From a Health Demography Approach. Int J Nephrol., 2022, 2022: 5511555. 10.1155/2022/5511555 PMC9135572 35634197

[81] Akokuwebe ME, Idemudia ES (2022). A community study of the risk factors and Perceived susceptibility to kidney Disease Risk in Lagos State, Southwest Nigeria. Afr J Biomed Res.

[82] Akokuwebe ME, Idemudia ES, Prevalence (2022). Incidence and perceived predisposition to kidney disease among nigerian Population Resident in Lagos State, Nigeria: A Community-based cross-sectional study. Afr J Biomed Res.

[83] Akokuwebe ME, Idemudia ES (2023). Prevalence and knowledge of kidney disease risk factors among Nigerians resident in Lagos State Metropolitan District, South West Nigeria. Ann Afr Med.

[84] Whitley R, Wang J-W, Fleury M-J, Liu A, Caron J (2017). Mental Health Status, Health Care Utilisation, and service satisfaction among immigrants in Montreal: an epidemiological comparison. Can J Psychiatry/La Revue Canadienne de Psychiatrie.

[85] Xiong MZ, Zhao P, Zou X, Hall B, Cao H, Wang C (2021). Health service utilisation among african migrants in China: a nationwide cross-sectional study. BMJ Open.

[86] Maslow AH (1943). A theory of human motivation. Psychol Rev.

[87] Quarshie E, Alagidede IP, Duodu A, Sosi ET (2022). Moonlighting behavior among migrants: determinants and implications for Wellbeing in South Africa. Afr Hum Mobil Rev.

[88] Shao Q (2022). Does less working time improve life satisfaction? Evidence from european Social Survey. Health Econ Rev.

[89] Møller V (1998). Quality of life in South Africa: post-apartheid trends. Soc Indic Res.

[90] Møller V, Radloff S (2010). Monitoring perceptions of social progress and pride of place in a south african community. Appl Res Qual Life.

[91] Ajaero CK, Ebimgbo S, Ezeibe C, Ugwu C, Nzeadibe C. Osabede, N. Life satisfaction in South Africa: the influence of inter-provincial Migration Status. Psychol Stud, 2023, 57.

[92] Metzner F, Adedeji A, Wichmann ML, Zaheer Z, Schneider L, Schlachzig L, Richters J, Heumann S, Mays D. Experiences of discrimination and everyday racism among children and adolescents with an immigrant background – results of a systematic literature review on the impact of discrimination on the Developmental Outcomes of Minors Worldwide. Front Psychol, 2022, 13. 10.3389/fpsyg.2022.805941 PMC9126147 35615177

[93] Loewe N, Bagherzadeh M, Araya-Castillo L, Thieme C, Batista-Foguet JM (2014). Life domain satisfactions as predictors of overall life satisfaction among workers: evidence from Chile. Soc Indic Res.

[94] Poppe A, Jirovsky E, Blacklock C, Laxmikanth P, Moosa S, De Maeseneer J, Kutalek R, Peersman W (2014). Why sub-saharan african health workers migrate to european countries that do not actively recruit: a qualitative study post-migration. Global Health Action.

[95] Esipova N, Ray J, Pugliese AG. World Poll: The Many Faces of Global Migration - Based on research in more than 150 countries. IOM Migration Research Series, International Organization for Migration (IOM) in collaboration with Gallup, 2011, 43, 1–76.

[96] He Z, Cheng Z, Bishwajit G, Zou D (2018). Wealth inequality as a predictor of Subjective Health, happiness and life satisfaction among nepalese women. Int J Environ Res Public Health.

[97] Sourtzi P, Galanis P, Konstantakopoulou O, Siskou O, Kaitelidou D (2020). Factors that influence the health status of immigrants living in Greece. AIMS Public Health.

[98] McKay S, Markova E, Paraskevopoulou A. Undocumented Workers’ Transitions: Legal Status, Migration, and Work in Europe. Routledge Advances in Sociology; 2011.

[99] Walther L, Fuchs LM, Schupp J, von Scheve C (2020). Living conditions and the Mental Health and Wellbeing of Refugees: evidence from a LargeScale german survey. J Immigr Minor Health.

[100] Fakhoury J, Burton-Jeangros C, Consoli L, Duvoisin A, Courvoisier D, Jackson Y (2021). Mental health of undocumented migrants and migrants undergoing regularization in Switzerland: a cross-sectional study. BMC Psychiatry.

[101] Consoli L, Burton-Jeangros C, Jackson Y (2022). When the set of known Opportunities Broadens: aspirations and imagined futures of undocumented Migrants applying for regularization. Swiss J Sociol.

[102] Blaauw PF, Pretorius M. “I am 30 and I have nothing”: The context of reception and the lived experiences of foreign migrants working as car guards in Johannesburg’s West Rand. GeoJournal, 2023; 88 (1), 121–135. 10.1007/s10708-022-10594-8 PMC8853150 35194298

[103] Biyase M, Fisher B, Pretorius M (2021). Remittances and subjective well-being: a static versus dynamic panel approach to happiness. Migration Lett.

[104] Malvaso A, Kang W. The relationship between areas of life satisfaction, personality, and overall life satisfaction: an integrated account. Front Psychol, 2022; *13*. 10.3389/fpsyg.2022.894610 PMC9532945 36211891

