# Supplementary material for: Determinants of life satisfaction among migrants in South Africa: an analysis of the GCRO’s quality of life survey (2009–2021)
Source: BMC Public Health. 2023 Oct 18;23:2030. doi: 10.1186/s12889-023-16868-1 (PMC10585904; doi:10.1186/s12889-023-16868-1)
Supplement: Supplementary file 3 — Supplementary Material 3 [file 12889_2023_16868_MOESM3_ESM.pdf]

```
clear

use "C:gcro-qlf-2009-v1.1-20011209-stata12\gcro-qlf-2009-v1.1-20011209-s12.dta", clear

*numlabel, add

*rename ID Unique_Identifier

*Weights

*Persons weight

* Weight

*Household weight

* HH_WEIGHT


*In-mighrants and Immigrants

ta Q1_9

ta Q1_11

generate Migration_Stat=.

replace Migration_Stat=1 if Q1_9==1 | Q1_9==2 | Q1_9==4 | Q1_9==5 | Q1_9==6 | Q1_9==7 |
Q1_9==8 | Q1_9==9

replace Migration_Stat=1 if Q1_11==1 | Q1_11==2 | Q1_11==4 | Q1_11==5 | Q1_11==6 | Q1_11==7
| Q1_11==8 | Q1_11==9

replace Migration_Stat=2 if Q1_9==10

replace Migration_Stat=2 if Q1_11==10

replace Migration_Stat=3 if Q1_9==3

replace Migration_Stat=3 if Q1_11==3

ta Migration_Stat

recode Migration_Stat (1=1 "In-migrants") (2=2 "Immigrants") (3=3 "Non_migrants"), gen
(Migration_Status)

replace Migration_Status=. if Migration_Status==3

ta Migration_Status [iw=Weight]


*Individual factors

ta Q12_2 [iw=Weight]

replace Q12_2=. if Q12_2==17

recode Q12_2 (18/27=1 "18-27") (28/37=2 "28-37") (38/47=3 "38-47") (48/92=4 "48+"), gen
(Grouped_Age)

ta Grouped_Age [iw=Weight]
```

```
ta A19 [iw=Weight]
ta Educ [iw=Weight]
recode Educ (1=1 "No_Edu") (2=2 "Primary") (3/4=3 "Secondary_Higher"), gen (Highest_Education)
ta Highest_Education [iw=Weight]
ta A18 [iw=Weight]
recode A18 (1=1 "Black_African") (2/4=2 "Non_Black_African"), gen (Population_group)
ta Population_group [iw=Weight]
ta Q12_12 [iw=Weight]
replace Q12_12=. if Q12_12==-1
replace Q12_12=. if Q12_12==13
recode Q12_12 (1=1 "No_income") (2/7=2 "Low") (8=3 "Middle") (9/12=4 "High"), gen (Income)
ta Income [iw=Weight]
ta Q7_1 [iw=Weight]
recode Q7_1 (1=2) (2=1)
*ta Empl
*Occupation
*ta Marrital_Status [iw=Weight]
*Access to media
*Parity/number of children
*IPV
ta Q10_3 [iw=Weight]
replace Q10_3=. if Q10_3==4
recode Q10_3 (3=1) (1/2=2)
ta Q10_1 [iw=Weight]
replace Q10_1=. if Q10_1>=4

*Household-level factors
*HH Wealth index
ta Q12_6 [iw=Weight]
ta Q12_5 [iw=Weight]
recode Q12_5 (1=1 "One") (2=2 "Two") (3=3 "Three") (4/26=4 "Four_More"), gen (People_in_HH)
ta People_in_HH [iw=Weight]
ta Q12_7 [iw=Weight]
recode Q12_7 (0=1) (1=2) (2=3) (3=4) (4/16=5)
```

```
*ta HH member aged 60+ [iw=Weight]

ta Q4_13 [iw=Weight],nol

recode Q4_13 (2=1) (1=2) (0=3)

ta Q12_13_3 [iw=Weight]

recode Q12_13_3 (1=2) (2=1)


*Community-level factors

gen Media=.

replace Media= 1 if Q4_9_1==2 & Q4_9_2==2 & Q4_9_4==2 & Q4_9_5==2 & Q4_9_6==2 & Q4_9_8==2

replace Media= 2 if Q4_9_1==1 & Q4_9_2==1 & Q4_9_4==1 & Q4_9_5==1 & Q4_9_6==1 & Q4_9_8==1

recode Media (1=1 "No") (2=2 "Yes"), gen (Media_Access)


*Residential status

ta A20 [iw=Weight]

recode A20 (1/6=1) (7/10=2), gen (Type_Dwelling)

ta Type_Dwelling [iw=Weight]

ta Migration_Status [iw=Weight]

ta Media_Access [iw=Weight]


ta Migration_Status [iw=Weight]


*Individual factors

ta Grouped_Age [iw=Weight]

ta A19 [iw=Weight]

ta Highest_Education [iw=Weight]

ta Population_group [iw=Weight]

ta Income [iw=Weight]

ta Q7_1 [iw=Weight]

*ta Empl

*Occupation

*ta Marrital_Status [iw=Weight]

*Access to media
```

```
*Parity/number of children
*IPV
ta Q10_3 [iw=Weight]
ta Q10_1 [iw=Weight]

*Household-level factors
*HH Wealth index
ta Q12_6 [iw=Weight]
ta People_in_HH [iw=Weight]
ta Q12_7 [iw=Weight]
*ta HH member aged 60+ [iw=Weight]
ta Q4_13 [iw=Weight]
ta Q12_13_3 [iw=Weight]

*Community-level factors

ta Media_Access
*Residential status
ta Type_Dwelling [iw=Weight]
ta Migration_Status [iw=Weight]
ta Media_Access [iw=Weight]

********************************************************************************
*Individual factors

ta Migration_Status
gen life_sati_5=Q6_9 if Migration_Status==1 | Migration_Status==2
*gen lifesatisfaction=LifeSati if Migration_Status==1 | Migration_Status==2
gen groupedage=Grouped_Age if Migration_Status==1 | Migration_Status==2
gen sex=A19 if Migration_Status==1 | Migration_Status==2
gen highesteducation=Highest_Education if Migration_Status==1 | Migration_Status==2
gen populationgroup=Population_group if Migration_Status==1 | Migration_Status==2
gen income=Income if Migration_Status==1 | Migration_Status==2
gen working=Q7_1 if Migration_Status==1 | Migration_Status==2
```

```
*gen marritalstatus=Marrital_Status if Migration_Status==1 | Migration_Status==2
gen medicalaid=Q10_3 if Migration_Status==1 | Migration_Status==2
gen healthfacility=Q10_1 if Migration_Status==1 | Migration_Status==2

*Household-level factors
gen hhhead=Q12_6 if Migration_Status==1 | Migration_Status==2
recode hhhead (1/9=1 "HhH"), gen (HHead)
ta HHead
gen HHeadSex=.
replace HHeadSex=1 if HHead==1 & sex==1
replace HHeadSex=2 if HHead==1 & sex==2
gen hhmembers=People_in_HH if Migration_Status==1 | Migration_Status==2
gen under18=Q12_7 if Migration_Status==1 | Migration_Status==2
*gen sixtyplus=q14_7_60plus_recode if Migration_Status==1 | Migration_Status==2
gen childhunger=Q4_13 if Migration_Status==1 | Migration_Status==2
gen social_grant=Q12_13_3 if Migration_Status==1 | Migration_Status==2

*Community-level factors
gen dwellingtype=Type_Dwelling if Migration_Status==1 | Migration_Status==2
*gen Migration_Status if Migration_Status==1 | Migration_Status==2
gen mediaaccess=Media_Access if Migration_Status==1 | Migration_Status==2

drop if Migration_Status==.
*drop if lifesatisfaction==.
drop if groupedage==.
drop if sex==.
drop if highesteducation==.
drop if populationgroup==.
drop if income==.
drop if working==.
*drop if marritalstatus==.
drop if medicalaid==.
drop if healthfacility==.
drop if dwellingtype==.
```

```
*Frequency
ta life_sati_5 Migration_Status [iw=Weight]
ta Migration_Status [iw=Weight]
*ta lifesatisfaction
ta groupedage Migration_Status [iw=Weight]
ta sex Migration_Status [iw=Weight]
ta highesteducation Migration_Status [iw=Weight]
ta populationgroup Migration_Status [iw=Weight]
ta income Migration_Status [iw=Weight]
ta working Migration_Status [iw=Weight]
*ta marritalstatus Migration_Status [iw=Weight]
ta medicalaid Migration_Status [iw=Weight]
ta healthfacility Migration_Status [iw=Weight]

*Household-level factors
ta HHeadSex Migration_Status [iw=Weight]
ta hhmembers Migration_Status [iw=Weight]
ta under18 Migration_Status [iw=Weight]
*ta sixtyplus Migration_Status [iw=Weight]
ta childhunger Migration_Status [iw=Weight]
ta social_grant Migration_Status [iw=Weight]

*Community-level factors
ta dwellingtype Migration_Status [iw=Weight]
ta Migration_Status Migration_Status [iw=Weight]
ta mediaaccess Migration_Status [iw=Weight]

*Frequency by sex
ta Migration_Status sex [iw=Weight]
ta life_sati_5 [iw=Weight]
ta life_sati_5 Migration_Status [iw=Weight]
ta life_sati_5 sex [iw=Weight]
ta groupedage sex [iw=Weight]
```

```
ta sex [iw=Weight]
ta highesteducation sex [iw=Weight]
ta populationgroup sex [iw=Weight]
ta income sex [iw=Weight]
ta working sex [iw=Weight]
*ta marritalstatus sex [iw=Weight]
ta medicalaid sex [iw=Weight]
ta healthfacility sex [iw=Weight]

*Household-level factors
ta HHeadSex [iw=Weight]
ta hhmembers sex [iw=Weight]
ta under18 sex [iw=Weight]
*ta sixtyplus sex [iw=Weight]
ta childhunger sex [iw=Weight]
ta social_grant sex [iw=Weight]

*Community-level factors
ta dwellingtype sex [iw=Weight]
ta Migration_Status sex [iw=Weight]
ta mediaaccess sex [iw=Weight]

**************************
table sex life_sati_5 Migration_Status [iw=Weight]
table life_sati_5 Migration_Status [iw=Weight]
```
