# Supplementary material for: Determinants of life satisfaction among migrants in South Africa: an analysis of the GCRO’s quality of life survey (2009–2021)
Source: BMC Public Health. 2023 Oct 18;23:2030. doi: 10.1186/s12889-023-16868-1 (PMC10585904; doi:10.1186/s12889-023-16868-1)
Supplement: Supplementary file 4 — Supplementary Material 4 [file 12889_2023_16868_MOESM4_ESM.pdf]

```
clear
use "C:\gcro-qls-2011-v1-stata12\Stata12\GCRO 2011_28Feb12noGIS.dta", clear

*numlabel, add
*rename recordid Unique_Identifier
*Weights
* WeightGCRO



*In-mighrants and Immigrants
ta A_2_3
recode A_2_3 (1/8=1 "In-migrants") (9=2 "Immigrants"), gen (Migration_Status)
ta Migration_Status [iw=WeightGCRO]

*Individual factors
ta A_12_2 [iw=WeightGCRO]
recode A_12_2  (16/17=0 "Ünder18") (18/27=1 "18-27") (28/37=2 "28-37") (38/47=3 "38-47") (48/99=4 "48+"), gen (Grouped_Age)
replace Grouped_Age=. if Grouped_Age==0
ta Grouped_Age [iw=WeightGCRO]
ta A2 [iw=WeightGCRO]
ta Education [iw=WeightGCRO]
recode Education (0=1 "No_Edu") (1=2 "Primary") (2/4=3 "Secondary_Higher"), gen (Highest_Education)
ta Highest_Education [iw=WeightGCRO]
ta A1 [iw=WeightGCRO]
recode A1 (1=1 "Black_African") (2/4=2 "Non_Black_African"), gen (Population_group)
ta Population_group [iw=WeightGCRO]
ta A_12_7 [iw=WeightGCRO]
replace A_12_7=. if A_12_7==13
recode A_12_7 (1=1 "No_Income") (2/7=2 "Low") (8=3 "Middle") (9/12=4 "High"), gen (Income)
ta Income [iw=WeightGCRO]
recode A_8_21 (0=1) (1=2)
*ta EmploymentStatus [iw=WeightGCRO]
```

*Occupation

*ta q14_4_rel_status [iw=WeightGCRO]

*replace q14_4_rel_status=. if q14_4_rel_status==7

*recode q14_4_rel_status (3=1 "Never_Married") (1=2 "Married_Cohab") (2=2) (6=2) (4=3 "Divorced") (5=4 "Widowed"), gen (Marrital_Status)

*ta Marrital_Status [iw=WeightGCRO]

*Access to media

*Parity/number of children

*IPV

ta A_11_3 [iw=WeightGCRO]

replace A_11_3=. if A_11_3==4

recode A_11_3 (3=1) (1/2=2)

ta A_11_1 [iw=WeightGCRO]

replace A_11_1=. if A_11_1>=4

*Household-level factors

*HH Wealth index

ta A_12_5 [iw=WeightGCRO]

ta A_12_4 [iw=WeightGCRO]

recode A_12_4 (1=1 "One") (2=2 "Two") (3=3 "Three") (4/18=4 "Four_More"), gen (People_in_HH)

ta People_in_HH [iw=WeightGCRO]

ta A_12_6 [iw=WeightGCRO]

recode A_12_6 (0=1) (1=2) (2=3) (3=4) (4/8=5)

*ta q14_7_60plus_recode [iw=WeightGCRO]

ta A_5_28 [iw=WeightGCRO]

recode A_5_28 (0=1) (1=2)

ta A_12_10 [iw=WeightGCRO]

recode A_12_10 (0=1) (1=2)

*Community-level factors

gen Media=.

replace Media= 1 if A_5_17==0 & A_5_18==0 & A_5_19==0 & A_5_20==0 & A_5_21==0 & A_5_22==0 & A_5_23==0

replace Media= 2 if A_5_17==1 & A_5_18==1 & A_5_19==1 & A_5_20==1 & A_5_21==1 & A_5_22==1 & A_5_23==1

```
recode Media (1=1 "No") (2=2 "Yes"), gen (Media_Access)

*Residential status
ta DwellingType [iw=WeightGCRO]
replace DwellingType=. if DwellingType==3
ta Migration_Status [iw=WeightGCRO]
ta Media_Access [iw=WeightGCRO]

ta Migration_Status [iw=WeightGCRO]

*Individual factors
ta Grouped_Age [iw=WeightGCRO]
ta A2 [iw=WeightGCRO]
ta Highest_Education [iw=WeightGCRO]
ta Population_group [iw=WeightGCRO]
ta Income [iw=WeightGCRO]
ta A_8_21 [iw=WeightGCRO]
ta A_11_3 [iw=WeightGCRO]
ta A_11_1 [iw=WeightGCRO]

*Household-level factors
*HH Wealth index
ta A_12_5 [iw=WeightGCRO]
ta People_in_HH [iw=WeightGCRO]
ta A_12_6 [iw=WeightGCRO]
*ta q14_7_60plus_recode [iw=WeightGCRO]
ta A_5_28 [iw=WeightGCRO]
ta A_12_10 [iw=WeightGCRO]

*Community-level factors
*Residential status
ta DwellingType [iw=WeightGCRO]
ta Migration_Status [iw=WeightGCRO]
ta Media_Access [iw=WeightGCRO]
```

```
*******************************************************************************
*Individual factors

ta Migration_Status
gen life_sati_5=A_7_10 if Migration_Status==1 | Migration_Status==2
*gen lifesatisfaction=LifeSati if Migration_Status==1 | Migration_Status==2
gen groupedage=Grouped_Age if Migration_Status==1 | Migration_Status==2
gen sex=A2 if Migration_Status==1 | Migration_Status==2
gen highesteducation=Highest_Education if Migration_Status==1 | Migration_Status==2
gen populationgroup=Population_group if Migration_Status==1 | Migration_Status==2
gen income=Income if Migration_Status==1 | Migration_Status==2
gen working=A_8_21 if Migration_Status==1 | Migration_Status==2
*gen marritalstatus=Marrital_Status if Migration_Status==1 | Migration_Status==2
gen medicalaid=A_11_3 if Migration_Status==1 | Migration_Status==2
gen healthfacility=A_11_1 if Migration_Status==1 | Migration_Status==2

*Household-level factors
gen hhhead=A_12_5 if Migration_Status==1 | Migration_Status==2
recode hhhead (1/9=1 "HhH"), gen (HHead)
ta HHead
gen HHeadSex=.
replace HHeadSex=1 if HHead==1 & sex==1
replace HHeadSex=2 if HHead==1 & sex==2
gen hhmembers=People_in_HH if Migration_Status==1 | Migration_Status==2
gen under18=A_12_6 if Migration_Status==1 | Migration_Status==2
*gen sixtyplus=q14_7_60plus_recode if Migration_Status==1 | Migration_Status==2
gen childhunger=A_5_28 if Migration_Status==1 | Migration_Status==2
gen social_grant=A_12_10 if Migration_Status==1 | Migration_Status==2

*Community-level factors
gen dwellingtype=DwellingType if Migration_Status==1 | Migration_Status==2
*gen Migration_Status if Migration_Status==1 | Migration_Status==2
gen mediaaccess=Media_Access if Migration_Status==1 | Migration_Status==2
```

```
drop if Migration_Status==.
*drop if lifesatisfaction==.
drop if groupedage==.
drop if sex==.
drop if highesteducation==.
drop if populationgroup==.
drop if income==.
drop if working==.
*drop if marritalstatus==.
drop if medicalaid==.
drop if healthfacility==.
drop if dwellingtype==.

*Frequency
ta life_sati_5 Migration_Status [iw=WeightGCRO]
ta Migration_Status [iw=WeightGCRO]
*ta lifesatisfaction
ta groupedage Migration_Status [iw=WeightGCRO]
ta sex Migration_Status [iw=WeightGCRO]
ta highesteducation Migration_Status [iw=WeightGCRO]
ta populationgroup Migration_Status [iw=WeightGCRO]
ta income Migration_Status [iw=WeightGCRO]
ta working Migration_Status [iw=WeightGCRO]
*ta marritalstatus Migration_Status [iw=WeightGCRO]
ta medicalaid Migration_Status [iw=WeightGCRO]
ta healthfacility Migration_Status [iw=WeightGCRO]

*Household-level factors
ta HHeadSex Migration_Status [iw=WeightGCRO]
ta hhmembers Migration_Status [iw=WeightGCRO]
ta under18 Migration_Status [iw=WeightGCRO]
*ta sixtyplus Migration_Status [iw=WeightGCRO]
```

```
ta childhunger Migration_Status [iw=WeightGCRO]
ta social_grant Migration_Status [iw=WeightGCRO]

*Community-level factors
ta dwellingtype Migration_Status [iw=WeightGCRO]
ta Migration_Status Migration_Status [iw=WeightGCRO]
ta mediaaccess Migration_Status [iw=WeightGCRO]

*Frequency by sex
ta Migration_Status sex [iw=WeightGCRO]
ta life_sati_5 [iw=WeightGCRO]
ta life_sati_5 Migration_Status [iw=WeightGCRO]
ta life_sati_5 sex [iw=WeightGCRO]
ta groupedage sex [iw=WeightGCRO]
ta sex [iw=WeightGCRO]
ta highesteducation sex [iw=WeightGCRO]
ta populationgroup sex [iw=WeightGCRO]
ta income sex [iw=WeightGCRO]
ta working sex [iw=WeightGCRO]
*ta marritalstatus sex [iw=WeightGCRO]
ta medicalaid sex [iw=WeightGCRO]
ta healthfacility sex [iw=WeightGCRO]

*Household-level factors
ta HHeadSex [iw=WeightGCRO]
ta hhmembers sex [iw=WeightGCRO]
ta under18 sex [iw=WeightGCRO]
*ta sixtyplus sex [iw=WeightGCRO]
ta childhunger sex [iw=WeightGCRO]
ta social_grant sex [iw=WeightGCRO]

*Community-level factors
ta dwellingtype sex [iw=WeightGCRO]
ta Migration_Status sex [iw=WeightGCRO]
```

ta mediaaccess sex [iw=WeightGCRO]

****

table sex life_sati_5 Migration_Status [iw=WeightGCRO]

table life_sati_5 Migration_Status [iw=WeightGCRO]
