# Supplementary material for: Determinants of life satisfaction among migrants in South Africa: an analysis of the GCRO’s quality of life survey (2009–2021)
Source: BMC Public Health. 2023 Oct 18;23:2030. doi: 10.1186/s12889-023-16868-1 (PMC10585904; doi:10.1186/s12889-023-16868-1)
Supplement: Supplementary file 5 — Supplementary Material 5 [file 12889_2023_16868_MOESM5_ESM.pdf]

```
clear

use "C:\qols-iii-2013-2014-v1-stata\qol-iii-2013-2014-v1.dta", clear

numlabel, add

*rename ID Unique_Identifier

*Weights

* GCROweight



*In-mighrants and Immigrants

ta X2_3

recode X2_3 (1=1 "In-migrants") (2=2 "Immigrants"), gen (Migration_Status)

ta Migration_Status [iw=GCROweight]


*Individual factors

ta X12_2 [iw=GCROweight]

recode X12_2  (14/17=0 "Ünder18") (18/27=1 "18-27") (28/37=2 "28-37") (38/47=3 "38-47")
(48/100=4 "48+"), gen (Grouped_Age)

replace Grouped_Age=. if Grouped_Age==0

ta Grouped_Age [iw=GCROweight]

ta H_2 [iw=GCROweight]

ta X12_1_RECODED [iw=GCROweight]

recode X12_1_RECODED (3=1 "No_Edu") (4=2 "Primary") (1/2=3 "Secondary_Higher") (5=3), gen
(Highest_Education)

ta Highest_Education [iw=GCROweight]

ta H_1 [iw=GCROweight]

replace H_1=. if H_1==5

recode H_1 (1=1 "Black_African") (2/4=2 "Non_Black_African"), gen (Population_group)

ta Population_group [iw=GCROweight]

ta X12_8 [iw=GCROweight]

replace X12_8=. if X12_8==18

recode X12_8 (17=1 "No_Income") (1/6=2 "Low") (7/8=3 "Middle") (9/16=4 "High"), gen (Income)
```

```
ta Income [iw=GCROweight]

recode X8_8 (0=1) (1=2)

*ta employment_status [iw=GCROweight]

**employment_status

*Occupation

*ta q14_4_rel_status [iw=GCROweight]

*replace q14_4_rel_status=. if q14_4_rel_status==7

*recode q14_4_rel_status (3=1 "Never_Married") (1=2 "Married_Cohab") (2=2) (6=2) (4=3 "Divorced") (5=4 "Widowed"), gen (Marrital_Status)

*ta Marrital_Status [iw=GCROweight]

*Access to media

*Parity/number of children

*IPV

ta X11_3 [iw=GCROweight]

replace X11_3=. if X11_3==4

recode X11_3 (3=1) (1/2=2)

ta X11_1 [iw=GCROweight]

replace X11_1=. if X11_1>=4


*Household-level factors

*HH Wealth index

ta X12_5 [iw=GCROweight]

ta X12_4_RECODED [iw=GCROweight]

recode X12_4_RECODED (1=1 "One") (2=2 "Two") (3=3 "Three") (4/7=4 "Four_More"), gen (People_in_HH)

ta People_in_HH [iw=GCROweight]

ta X12_6 [iw=GCROweight]

recode X12_6  (0=1) (1=2) (2=3) (3=4) (4/18=5)

*ta q14_7_60plus_recode [iw=GCROweight]

ta X5_8 [iw=GCROweight]

recode X5_8 (0=1) (1=2) (2=3)

ta X12_11 [iw=GCROweight]

recode X12_11 (0=1) (1=2)
```

```
*Community-level factors

gen Media=.

replace Media= 1 if X5_4_1==-1 & X5_4_2==-1 & X5_4_3==-1 & X5_4_4==-1 & X5_4_5==-1 & X5_4_6==-1 & X5_4_7==-1 & X5_6_2==-1 & X5_6_6==-1

replace Media= 2 if X5_4_1==1 & X5_4_2==2 & X5_4_3==3 & X5_4_4==4 & X5_4_5==5 & X5_4_6==6 & X5_4_7==7 & X5_6_2==2 & X5_6_6==6

*Q5_1 X5_6_2 X5_6_6

recode Media (1=1 "No") (2=2 "Yes"), gen (Media_Access)


*Residential status

ta H_3_RECODED [iw=GCROweight]

replace H_3_RECODED=. if H_3_RECODED==3

ta Migration_Status [iw=GCROweight]

ta Media_Access [iw=GCROweight]


ta Migration_Status [iw=GCROweight]


*Individual factors

ta Grouped_Age [iw=GCROweight]

ta H_2 [iw=GCROweight]

ta Highest_Education [iw=GCROweight]

ta Population_group [iw=GCROweight]

ta Income [iw=GCROweight]

ta X8_8 [iw=GCROweight]

ta X11_3 [iw=GCROweight]

ta X11_1 [iw=GCROweight]


*Household-level factors

*HH Wealth index

ta X12_5 [iw=GCROweight]

ta People_in_HH [iw=GCROweight]

ta X12_6 [iw=GCROweight]

ta X5_8 [iw=GCROweight]

ta X12_11 [iw=GCROweight]
```

```
*Community-level factors
*Residential status
ta H_3_RECODED [iw=GCROweight]
ta Migration_Status [iw=GCROweight]
ta Media_Access [iw=GCROweight]

******************************************************************************
*Individual factors

ta Migration_Status
gen life_sati_5=X7_10 if Migration_Status==1 | Migration_Status==2
*gen lifesatisfaction=LifeSati if Migration_Status==1 | Migration_Status==2
gen groupedage=Grouped_Age if Migration_Status==1 | Migration_Status==2
gen sex=H_2 if Migration_Status==1 | Migration_Status==2
gen highesteducation=Highest_Education if Migration_Status==1 | Migration_Status==2
gen populationgroup=Population_group if Migration_Status==1 | Migration_Status==2
gen income=Income if Migration_Status==1 | Migration_Status==2
gen working=X8_8 if Migration_Status==1 | Migration_Status==2
*gen marritalstatus=Marrital_Status if Migration_Status==1 | Migration_Status==2
gen medicalaid=X11_3 if Migration_Status==1 | Migration_Status==2
gen healthfacility=X11_1 if Migration_Status==1 | Migration_Status==2

*Household-level factors
gen hhhead=X12_5 if Migration_Status==1 | Migration_Status==2
recode hhhead (1/9=1 "HhH"), gen (HHead)
ta HHead
gen HHeadSex=.
replace HHeadSex=1 if HHead==1 & sex==1
replace HHeadSex=2 if HHead==1 & sex==2
gen hhmembers=People_in_HH if Migration_Status==1 | Migration_Status==2
gen under18=X12_6 if Migration_Status==1 | Migration_Status==2
*gen sixtyplus=q14_7_60plus_recode if Migration_Status==1 | Migration_Status==2
gen childhunger=X5_8 if Migration_Status==1 | Migration_Status==2
```

```
gen social_grant=X12_11 if Migration_Status==1 | Migration_Status==2

*Community-level factors
gen dwellingtype=H_3_RECODED if Migration_Status==1 | Migration_Status==2
*gen Migration_Status if Migration_Status==1 | Migration_Status==2
gen mediaaccess=Media_Access if Migration_Status==1 | Migration_Status==2



drop if Migration_Status==.
*drop if lifesatisfaction==.
drop if groupedage==.
drop if sex==.
drop if highesteducation==.
drop if populationgroup==.
drop if income==.
drop if working==.
*drop if marritalstatus==.
drop if medicalaid==.
drop if healthfacility==.
drop if dwellingtype==.

*Frequency
ta life_sati_5 Migration_Status [iw=GCROweight]
ta Migration_Status [iw=GCROweight]
*ta lifesatisfaction
ta groupedage Migration_Status [iw=GCROweight]
ta sex Migration_Status [iw=GCROweight]
ta highesteducation Migration_Status [iw=GCROweight]
ta populationgroup Migration_Status [iw=GCROweight]
ta income Migration_Status [iw=GCROweight]
ta working Migration_Status [iw=GCROweight]
*ta marritalstatus Migration_Status [iw=GCROweight]
ta medicalaid Migration_Status [iw=GCROweight]
ta healthfacility Migration_Status [iw=GCROweight]
```

```
*Household-level factors
ta HHeadSex Migration_Status [iw=GCROweight]
ta hhmembers Migration_Status [iw=GCROweight]
ta under18 Migration_Status [iw=GCROweight]
*ta sixtyplus Migration_Status [iw=GCROweight]
ta childhunger Migration_Status [iw=GCROweight]
ta social_grant Migration_Status [iw=GCROweight]

*Community-level factors
ta dwellingtype Migration_Status [iw=GCROweight]
ta Migration_Status Migration_Status [iw=GCROweight]
ta mediaaccess Migration_Status [iw=GCROweight]


*Frequency by sex
ta Migration_Status sex [iw=GCROweight]
ta life_sati_5 [iw=GCROweight]
ta life_sati_5 Migration_Status [iw=GCROweight]
ta life_sati_5 sex [iw=GCROweight]
ta groupedage sex [iw=GCROweight]
ta sex [iw=GCROweight]
ta highesteducation sex [iw=GCROweight]
ta populationgroup sex [iw=GCROweight]
ta income sex [iw=GCROweight]
ta working sex [iw=GCROweight]
*ta marritalstatus sex [iw=GCROweight]
ta medicalaid sex [iw=GCROweight]
ta healthfacility sex [iw=GCROweight]

*Household-level factors
ta HHeadSex [iw=GCROweight]
ta hhmembers sex [iw=GCROweight]
ta under18 sex [iw=GCROweight]
```

*ta sixtyplus sex [iw=GCROweight]

ta childhunger sex [iw=GCROweight]

ta social_grant sex [iw=GCROweight]

*Community-level factors

ta dwellingtype sex [iw=GCROweight]

ta Migration_Status sex [iw=GCROweight]

ta mediaaccess sex [iw=GCROweight]

****

table sex life_sati_5 Migration_Status [iw=GCROweight]

table life_sati_5 Migration_Status [iw=GCROweight]
