# Supplementary material for: Determinants of life satisfaction among migrants in South Africa: an analysis of the GCRO’s quality of life survey (2009–2021)
Source: BMC Public Health. 2023 Oct 18;23:2030. doi: 10.1186/s12889-023-16868-1 (PMC10585904; doi:10.1186/s12889-023-16868-1)
Supplement: Supplementary file 6 — Supplementary Material 6 [file 12889_2023_16868_MOESM6_ESM.pdf]

```
clear
use "C:\qol-iv-2015-2016-v1-stata\qol-iv-2015-2016-v1-stata\qol-iv-2014-2015-v1.dta", clear
numlabel, add
*rename ID Unique_Identifier

*Weights
*Bench_wgt_30002

*In-mighrants and Immigrants
ta Q2_1
replace Q2_1=. if Q2_1==1
recode Q2_1 (2=1 "In-migrants") (3=2 "Immigrants"), gen (Migration_Status)
ta Migration_Status [iw=Bench_wgt_30002]

*Individual factors
ta Q12_3 [iw=Bench_wgt_30002]
recode Q12_3 (18/27=1 "18-27") (28/37=2 "28-37") (38/47=3 "38-47") (48/100=4 "48+"), gen
(Grouped_Age)
ta Grouped_Age [iw=Bench_wgt_30002]
ta S8 [iw=Bench_wgt_30002]
ta Q12_1_Recode [iw=Bench_wgt_30002]
replace Q12_1_Recode=. if Q12_1_Recode==6
recode Q12_1_Recode (1=1 "No_Edu") (2=2 "Primary") (3/5=3 "Secondary_Higher"), gen
(Highest_Education)
ta Highest_Education [iw=Bench_wgt_30002]
ta S7 [iw=Bench_wgt_30002]
replace S7=. if S7==5
recode S7 (1=1 "Black_African") (2/4=2 "Non_Black_African"), gen (Population_group)
ta Population_group [iw=Bench_wgt_30002]
ta Q12_10 [iw=Bench_wgt_30002]
replace Q12_10=. if Q12_10==19
recode Q12_10 (17=1 "No_Income") (1/6=2 "Low") (7/8=3 "Middle") (9/16=4 "High"), gen (Income)
```

```
ta Income [iw=Bench_wgt_30002]

recode Q8_15 (0=1) (1=2)

ta Q8_15 [iw=Bench_wgt_30002]

**Employment_Status

*Occupation

*ta q14_4_rel_status [iw=Bench_wgt_30002]

*replace q14_4_rel_status=. if q14_4_rel_status==7

*recode q14_4_rel_status (3=1 "Never_Married") (1=2 "Married_Cohab") (2=2) (6=2) (4=3
"Divorced") (5=4 "Widowed"), gen (Marrital_Status)

*ta Marrital_Status [iw=Bench_wgt_30002]

*Access to media

*Parity/number of children

*IPV

ta Q11_6 [iw=Bench_wgt_30002]

replace Q11_6=. if Q11_6>=5

recode Q11_6 (4=1) (2/3=2)

ta Q11_1 [iw=Bench_wgt_30002]

replace Q11_1=. if Q11_1>=4


*Household-level factors

*HH Wealth index

ta Q12_6 [iw=Bench_wgt_30002]

ta Q12_5_Recode [iw=Bench_wgt_30002]

recode Q12_5_Recode (1=1 "One") (2=2 "Two") (3=3 "Three") (4/7=4 "Four_More"), gen
(People_in_HH)

ta People_in_HH [iw=Bench_wgt_30002]

ta Q12_7_Recode [iw=Bench_wgt_30002]

recode Q12_7_Recode (1=1) (2=2) (3=3) (4/11=4)

*ta q14_7_60plus_recode [iw=Bench_wgt_30002]

ta Q5_9 [iw=Bench_wgt_30002]

recode Q5_9 (1=1) (2/5=2) (6=3)

ta Q12_8 [iw=Bench_wgt_30002]

recode Q12_8 (0=1) (1=2)
```

```
*Community-level factors
gen Media=.
replace Media= 1 if Q5_7_1==0 & Q5_7_2==0 & Q5_7_3==0 & Q5_7_4==0 & Q5_7_5==0 & Q5_7_6==0 & Q5_7_7==0
replace Media= 2 if Q5_7_1==1 & Q5_7_2==1 & Q5_7_3==1 & Q5_7_4==1 & Q5_7_5==1 & Q5_7_6==1 & Q5_7_7==1
*Q5_1 Q5_2_2 Q5_2_6 Q5_2_7
recode Media (1=1 "No") (2=2 "Yes"), gen (Media_Access)

*Residential status
ta Q1_Recode_Dwelling [iw=Bench_wgt_30002]
replace Q1_Recode_Dwelling=. if Q1_Recode_Dwelling==3
ta Migration_Status [iw=Bench_wgt_30002]
ta Media_Access [iw=Bench_wgt_30002]


ta Migration_Status [iw=Bench_wgt_30002]

ta Q12_3 [iw=Bench_wgt_30002]
ta Grouped_Age [iw=Bench_wgt_30002]
ta S8 [iw=Bench_wgt_30002]
ta Q12_1_Recode [iw=Bench_wgt_30002]
ta Highest_Education [iw=Bench_wgt_30002]
ta Population_group [iw=Bench_wgt_30002]
ta Income [iw=Bench_wgt_30002]
ta Q8_15 [iw=Bench_wgt_30002]
**Employment_Status
*ta q14_4_rel_status [iw=weight]
*ta Marrital_Status [iw=weight]
ta Q11_6 [iw=Bench_wgt_30002]
ta Q11_1 [iw=Bench_wgt_30002]

*Household-level factors
```

```
ta Q12_6 [iw=Bench_wgt_30002]
ta Q12_5_Recode [iw=Bench_wgt_30002]
ta People_in_HH [iw=Bench_wgt_30002]
ta Q12_7_Recode [iw=Bench_wgt_30002]
*ta q14_7_60plus_recode [iw=Bench_wgt_30002]
ta Q5_9 [iw=Bench_wgt_30002]
ta Q12_8 [iw=Bench_wgt_30002]

*Community-level factors

ta Q1_Recode_Dwelling [iw=Bench_wgt_30002]
ta Migration_Status [iw=Bench_wgt_30002]
ta Media_Access [iw=Bench_wgt_30002]

********************************************************************************
*Individual factors

ta Migration_Status
gen life_sati_5=Q7_9 if Migration_Status==1 | Migration_Status==2
*gen lifesatisfaction=LifeSati if Migration_Status==1 | Migration_Status==2
gen groupedage=Grouped_Age if Migration_Status==1 | Migration_Status==2
gen sex=S8 if Migration_Status==1 | Migration_Status==2
gen highesteducation=Highest_Education if Migration_Status==1 | Migration_Status==2
gen populationgroup=Population_group if Migration_Status==1 | Migration_Status==2
gen income=Income if Migration_Status==1 | Migration_Status==2
gen working=Q8_15 if Migration_Status==1 | Migration_Status==2
*gen marritalstatus=Marrital_Status if Migration_Status==1 | Migration_Status==2
gen medicalaid=Q11_6 if Migration_Status==1 | Migration_Status==2
gen healthfacility=Q11_1 if Migration_Status==1 | Migration_Status==2

*Household-level factors
gen hhhead=Q12_6 if Migration_Status==1 | Migration_Status==2
recode hhhead (1/9=1 "HhH"), gen (HHead)
```

```
ta HHead
gen HHeadSex=.
replace HHeadSex=1 if HHead==1 & sex==1
replace HHeadSex=2 if HHead==1 & sex==2
gen hhmembers=People_in_HH if Migration_Status==1 | Migration_Status==2
gen under18=Q12_7_Recode if Migration_Status==1 | Migration_Status==2
*gen sixtyplus=q14_7_60plus_recode if Migration_Status==1 | Migration_Status==2
gen childhunger=Q5_9 if Migration_Status==1 | Migration_Status==2
gen social_grant=Q12_8 if Migration_Status==1 | Migration_Status==2

*Community-level factors
gen dwellingtype=Q1_Recode_Dwelling if Migration_Status==1 | Migration_Status==2
*gen Migration_Status if Migration_Status==1 | Migration_Status==2
gen mediaaccess=Media_Access if Migration_Status==1 | Migration_Status==2


drop if Migration_Status==.
*drop if lifesatisfaction==.
drop if groupedage==.
drop if sex==.
drop if highesteducation==.
drop if populationgroup==.
drop if income==.
drop if working==.
*drop if marritalstatus==.
drop if medicalaid==.
drop if healthfacility==.
drop if dwellingtype==.

*Frequency
ta life_sati_5 Migration_Status [iw=Bench_wgt_30002]
ta Migration_Status [iw=Bench_wgt_30002]
*ta lifesatisfaction
ta groupedage Migration_Status [iw=Bench_wgt_30002]
```

ta sex Migration_Status [iw=Bench_wgt_30002]

ta highesteducation Migration_Status [iw=Bench_wgt_30002]

ta populationgroup Migration_Status [iw=Bench_wgt_30002]

ta income Migration_Status [iw=Bench_wgt_30002]

ta working Migration_Status [iw=Bench_wgt_30002]

*ta marritalstatus Migration_Status [iw=Bench_wgt_30002]

ta medicalaid Migration_Status [iw=Bench_wgt_30002]

ta healthfacility Migration_Status [iw=Bench_wgt_30002]

*Household-level factors

ta HHeadSex Migration_Status [iw=Bench_wgt_30002]

ta hhmembers Migration_Status [iw=Bench_wgt_30002]

ta under18 Migration_Status [iw=Bench_wgt_30002]

*ta sixtyplus Migration_Status [iw=Bench_wgt_30002]

ta childhunger Migration_Status [iw=Bench_wgt_30002]

ta social_grant Migration_Status [iw=Bench_wgt_30002]

*Community-level factors

ta dwellingtype Migration_Status [iw=Bench_wgt_30002]

ta Migration_Status Migration_Status [iw=Bench_wgt_30002]

ta mediaaccess Migration_Status [iw=Bench_wgt_30002]

*Frequency by sex

ta Migration_Status [iw=Bench_wgt_30002]

ta life_sati_5 [iw=Bench_wgt_30002]

ta life_sati_5 Migration_Status [iw=Bench_wgt_30002]

ta life_sati_5 sex [iw=Bench_wgt_30002]

ta groupedage sex [iw=Bench_wgt_30002]

ta sex [iw=Bench_wgt_30002]

ta highesteducation sex [iw=Bench_wgt_30002]

ta populationgroup sex [iw=Bench_wgt_30002]

ta income sex [iw=Bench_wgt_30002]

```
ta working sex [iw=Bench_wgt_30002]
*ta marritalstatus sex [iw=Bench_wgt_30002]
ta medicalaid sex [iw=Bench_wgt_30002]
ta healthfacility sex [iw=Bench_wgt_30002]

*Household-level factors
ta HHeadSex [iw=Bench_wgt_30002]
ta hhmembers sex [iw=Bench_wgt_30002]
ta under18 sex [iw=Bench_wgt_30002]
*ta sixtyplus sex [iw=Bench_wgt_30002]
ta childhunger sex [iw=Bench_wgt_30002]
ta social_grant sex [iw=Bench_wgt_30002]

*Community-level factors
ta dwellingtype sex [iw=Bench_wgt_30002]
ta Migration_Status sex [iw=Bench_wgt_30002]
ta mediaaccess sex [iw=Bench_wgt_30002]

*******************************************
table sex life_sati_5 Migration_Status [iw=Bench_wgt_30002]
table life_sati_5 Migration_Status [iw=Bench_wgt_30002]
```
