# Supplementary material for: Determinants of life satisfaction among migrants in South Africa: an analysis of the GCRO’s quality of life survey (2009–2021)
Source: BMC Public Health. 2023 Oct 18;23:2030. doi: 10.1186/s12889-023-16868-1 (PMC10585904; doi:10.1186/s12889-023-16868-1)
Supplement: Supplementary file 7 — Supplementary Material 7 [file 12889_2023_16868_MOESM7_ESM.pdf]

```
clear

use "C:\gcro-qols-v-v1.1\qols-v-2017-2018-v1.1.dta", clear

numlabel, add

*rename ID Unique_Identifier

*Weights

*Persons weight

* DOWNSCALE_MUN_PP_BENCHWGT

*Household weight

* HH_WEIGHT


*In-mighrants and Immigrants

ta Q3_01_recode

replace Q3_01_recode=. if Q3_01_recode==1

recode Q3_01_recode (2=1 "In-migrants") (3=2 "Immigrants"), gen (Migration_Status)

ta Migration_Status [iw=weight]


*Outcome variable Life satisfaction

*ta F4lifsat

*ta F4lifsat,nol

*recode F4lifsat (0/4.999999999=0 "Suffering") (5/6.9999999=1 "Struggling") (7.002461/10=2 "Thriving"), gen (LifeSati)

*recode LifeSati (5=1)

*ta LifeSati



*Individual factors

ta Q15_02_age [iw=weight]

recode Q15_02_age (18/27=1 "18-27") (28/37=2 "28-37") (38/47=3 "38-47") (48/105=4 "48+"), gen (Grouped_Age)

ta Grouped_Age [iw=weight]

ta A2_Sex [iw=weight]

ta Q15_01_education_recode [iw=weight]

replace Q15_01_education_recode=. if Q15_01_education_recode==6
```

```
recode Q15_01_education_recode (1=1 "No_Edu") (2=2 "Primary") (3/5=3 "Secondary_Higher"), gen (Highest_Education)

ta Highest_Education [iw=weight]

ta A1_Pop_group [iw=weight]

replace A1_Pop_group=. if A1_Pop_group==5

recode A1_Pop_group (1=1 "Black_African") (2/4=2 "Non_Black_African"), gen (Population_group)

ta Population_group [iw=weight]

ta Q15_20_income [iw=weight]

replace Q15_20_income=. if Q15_20_income==18

recode Q15_20_income (17=1 "No_Income") (1/6=2 "Low") (7/8=3 "Middle") (9/16=4 "High"), gen (Income)

ta Income [iw=weight]

recode Q11_12_working (0=1) (1=2)

ta Q11_12_working [iw=weight]

*Occupation

*ta q14_4_rel_status [iw=weight]

*replace q14_4_rel_status=. if q14_4_rel_status==7

*recode q14_4_rel_status (3=1 "Never_Married") (1=2 "Married_Cohab") (2=2) (6=2) (4=3 "Divorced") (5=4 "Widowed"), gen (Marrital_Status)

*ta Marrital_Status [iw=weight]

*Access to media

*Parity/number of children

*IPV

ta Q14_04_medaid [iw=weight]

replace Q14_04_medaid=. if Q14_04_medaid>=5

recode Q14_04_medaid (4=1) (2/3=2)

ta Q14_01_healthcare_services [iw=weight]

replace Q14_01_healthcare_services=. if Q14_01_healthcare_services>=4


*Household-level factors

*HH Wealth index

ta Q15_05_head [iw=weight]

ta Q1_01_02_people [iw=weight]

recode Q1_01_02_people (1=1 "One") (2=2 "Two") (3=3 "Three") (4/30=4 "Four_More"), gen (People_in_HH)
```

```
*(1/3=1 "1-3") (4/5=2 "4-5") (6/7=3 "6more"), gen (People_in_HH)

ta People_in_HH [iw=weight]

ta Q15_09_under18 [iw=weight]

recode Q15_09_under18 (0=1) (1=2) (2=3) (3=4) (4/12=5)

*ta q14_7_60plus_recode [iw=weight]

ta Q7_05_child [iw=weight]

recode Q7_05_child (2=1) (1=2) (3=3)

ta Q15_12_grant [iw=weight]

recode Q15_12_grant (0=1) (1=2)


*Community-level factors

gen Media=.

replace Media= 1 if Q7_03_1==0 & Q7_03_2==0 & Q7_03_3==0 & Q7_03_4==0 & Q7_03_5==0 & Q7_03_6==0

replace Media= 2 if Q7_03_1==1 & Q7_03_2==1 & Q7_03_3==1 & Q7_03_4==1 & Q7_03_5==1 & Q7_03_6==1

recode Media (1=1 "No") (2=2 "Yes"), gen (Media_Access)

*Q6_02_2  Q6_02_6  Q6_02_7



*Residential status

ta A3_dwelling_recode [iw=weight]

replace A3_dwelling_recode=. if A3_dwelling_recode==3

ta Migration_Status [iw=weight]

ta Media_Access [iw=weight]



ta Migration_Status [iw=weight]


*Individual factors

ta Grouped_Age [iw=weight]

ta A2_Sex [iw=weight]

ta Q15_01_education_recode [iw=weight]

ta Highest_Education [iw=weight]
```

```
ta Population_group [iw=weight]
ta Income [iw=weight]
ta Q11_12_working [iw=weight]
*ta q14_4_rel_status [iw=weight]
*ta Marrital_Status [iw=weight]
ta Q14_04_medaid [iw=weight]
ta Q14_01_healthcare_services [iw=weight]


*Household-level factors
ta Q15_05_head [iw=weight]
ta People_in_HH [iw=weight]
ta Q15_09_under18 [iw=weight]
*ta q14_7_60plus_recode [iw=weight]
ta Q7_05_child [iw=weight]
ta Q15_12_grant [iw=weight]


*Community-level factors
ta A3_dwelling_recode [iw=weight]
ta Migration_Status [iw=weight]
ta Media_Access [iw=weight]



********************************************************************************
*Individual factors

ta Migration_Status
gen life_sati_5=Q10_10_life if Migration_Status==1 | Migration_Status==2
*gen lifesatisfaction=LifeSati if Migration_Status==1 | Migration_Status==2
gen groupedage=Grouped_Age if Migration_Status==1 | Migration_Status==2
gen sex=A2_Sex if Migration_Status==1 | Migration_Status==2
gen highesteducation=Highest_Education if Migration_Status==1 | Migration_Status==2
```

```
gen populationgroup=Population_group if Migration_Status==1 | Migration_Status==2
gen income=Income if Migration_Status==1 | Migration_Status==2
gen working=Q11_12_working if Migration_Status==1 | Migration_Status==2
*gen marritalstatus=Marrital_Status if Migration_Status==1 | Migration_Status==2
gen medicalaid=Q14_04_medaid if Migration_Status==1 | Migration_Status==2
gen healthfacility=Q14_01_healthcare_services if Migration_Status==1 | Migration_Status==2

*Household-level factors
gen hhhead=Q15_05_head if Migration_Status==1 | Migration_Status==2
recode hhhead (1/9=1 "HhH"), gen (HHead)
ta HHead
gen HHeadSex=.
replace HHeadSex=1 if HHead==1 & sex==1
replace HHeadSex=2 if HHead==1 & sex==2
gen hhmembers=People_in_HH if Migration_Status==1 | Migration_Status==2
gen under18=Q15_09_under18 if Migration_Status==1 | Migration_Status==2
*gen sixtyplus=q14_7_60plus_recode if Migration_Status==1 | Migration_Status==2
gen childhunger=Q7_05_child if Migration_Status==1 | Migration_Status==2
gen social_grant=Q15_12_grant if Migration_Status==1 | Migration_Status==2

*Community-level factors
gen dwellingtype=A3_dwelling_recode if Migration_Status==1 | Migration_Status==2
*gen Migration_Status if Migration_Status==1 | Migration_Status==2
gen mediaaccess=Media_Access if Migration_Status==1 | Migration_Status==2


drop if Migration_Status==.
*drop if lifesatisfaction==.
drop if groupedage==.
drop if sex==.
drop if highesteducation==.
drop if populationgroup==.
drop if income==.
drop if working==.
```

```
*drop if marritalstatus==.
drop if medicalaid==.
drop if healthfacility==.
drop if dwellingtype==.

*****************************************************************************************
*Frequency by migration status
ta life_sati_5 Migration_Status [iw=weight]
ta Migration_Status [iw=weight]
*ta lifesatisfaction Migration_Status [iw=weight]
*ta lifesatisfaction sex [iw=weight]
ta groupedage Migration_Status [iw=weight]
ta sex Migration_Status [iw=weight]
ta highesteducation Migration_Status [iw=weight]
ta populationgroup Migration_Status [iw=weight]
ta income Migration_Status [iw=weight]
ta working Migration_Status [iw=weight]
*ta marritalstatus Migration_Status [iw=weight]
ta medicalaid Migration_Status [iw=weight]
ta healthfacility Migration_Status [iw=weight]

*Household-level factors
ta HHeadSex Migration_Status [iw=weight]
ta hhmembers Migration_Status [iw=weight]
ta under18 Migration_Status [iw=weight]
*ta sixtyplus Migration_Status [iw=weight]
ta childhunger Migration_Status [iw=weight]
ta social_grant Migration_Status [iw=weight]

*Community-level factors
ta dwellingtype Migration_Status [iw=weight]
ta Migration_Status Migration_Status [iw=weight]
ta mediaaccess Migration_Status [iw=weight]
```

```
*Frequency by sex
ta Migration_Status [iw=weight]
ta life_sati_5 [iw=weight]
ta life_sati_5 Migration_Status [iw=weight]
ta life_sati_5 sex [iw=weight]
ta groupedage sex [iw=weight]
ta sex [iw=weight]
ta highesteducation sex [iw=weight]
ta populationgroup sex [iw=weight]
ta income sex [iw=weight]
ta working sex [iw=weight]
*ta marritalstatus sex [iw=weight]
ta medicalaid sex [iw=weight]
ta healthfacility sex [iw=weight]

*Household-level factors
ta HHeadSex sex [iw=weight]
ta hhmembers sex [iw=weight]
ta under18 sex [iw=weight]
*ta sixtyplus sex [iw=weight]
ta childhunger sex [iw=weight]
ta social_grant sex [iw=weight]

*Community-level factors
ta dwellingtype sex [iw=weight]
ta Migration_Status sex [iw=weight]
ta mediaaccess sex [iw=weight]

******************************************
table sex life_sati_5 Migration_Status [iw=weight]
table life_sati_5 Migration_Status [iw=weight]
```
