# Supplementary material for: Determinants of life satisfaction among migrants in South Africa: an analysis of the GCRO’s quality of life survey (2009–2021)
Source: BMC Public Health. 2023 Oct 18;23:2030. doi: 10.1186/s12889-023-16868-1 (PMC10585904; doi:10.1186/s12889-023-16868-1)
Supplement: Supplementary file 8 — Supplementary Material 8 [file 12889_2023_16868_MOESM8_ESM.pdf]

```
clear
use "C:\qols-2020-2021-v1\qols-2020-2021-new-weights-v1.dta", clear

numlabel, add
*Uniqu identifier
*rename unique_id Unique_Identifier
*Weights
*Persons weight
* DOWNSCALE_MUN_PP_BENCHWGT
*Household weight
* HH_WEIGHT

*In-mighrants and Immigrants
ta q3_1_birth_prov_recode
replace q3_1_birth_prov_recode=. if q3_1_birth_prov_recode==1
recode q3_1_birth_prov_recode (2=1 "In-migrants") (3=2 "Immigrants"), gen (Migration_Status)
ta Migration_Status [iw=DOWNSCALE_MUN_PP_BENCHWGT]

*Outcome variable Life satisfaction
ta q9_9_life
rename q9_9_life Life_Satisfaction
ta Life_Satisfaction

ta F4lifsat
ta F4lifsat,nol
recode F4lifsat (0/4.999999999=0 "Suffering") (5/6.9999999=1 "Struggling") (7.002461/10=2 "Thriving"), gen (LifeSati)
recode LifeSati (5=1)
ta LifeSati

*Individual factors
ta q14_2_age_topcode [iw=DOWNSCALE_MUN_PP_BENCHWGT]
```

```
recode q14_2_age_topcode (18/27=1 "18-27") (28/37=2 "28-37") (38/47=3 "38-47") (48/80=4 "48+"), gen (Grouped_Age)

ta Grouped_Age [iw=DOWNSCALE_MUN_PP_BENCHWGT]

ta a2_sex [iw=DOWNSCALE_MUN_PP_BENCHWGT]

ta q14_1_education_recode [iw=DOWNSCALE_MUN_PP_BENCHWGT]

replace q14_1_education_recode=. if q14_1_education_recode==6

recode q14_1_education_recode (1=1 "No_Edu") (2=2 "Primary") (3/5=3 "Secondary_Higher"), gen (Highest_Education)

ta Highest_Education [iw=DOWNSCALE_MUN_PP_BENCHWGT]

ta a1_pop_group [iw=DOWNSCALE_MUN_PP_BENCHWGT]

replace a1_pop_group=. if a1_pop_group==5

recode a1_pop_group (1=1 "Black_African") (2/4=2 "Non_Black_African"), gen (Population_group)

ta Population_group [iw=DOWNSCALE_MUN_PP_BENCHWGT]

ta q15_3_income [iw=DOWNSCALE_MUN_PP_BENCHWGT]

replace q15_3_income=. if q15_3_income>=18

recode q15_3_income (17=1 "no_inc") (1/6=2 "Low") (7/8=3 "Middle") (9/16=4 "High"), gen (Income)

ta Income [iw=DOWNSCALE_MUN_PP_BENCHWGT]

ta q10_2_working [iw=DOWNSCALE_MUN_PP_BENCHWGT]

recode q10_2_working (0=1) (1=2)

*Occupation

ta q14_4_rel_status [iw=DOWNSCALE_MUN_PP_BENCHWGT]

replace q14_4_rel_status=. if q14_4_rel_status==7

recode q14_4_rel_status (3=1 "Never_Married") (1=2 "Married_Cohab") (2=2) (6=2) (4=3 "Divorced") (5=4 "Widowed"), gen (Marrital_Status)

ta Marrital_Status [iw=DOWNSCALE_MUN_PP_BENCHWGT]

*Access to media

*Parity/number of children

*IPV

ta q13_5_medical_aid [iw=DOWNSCALE_MUN_PP_BENCHWGT]

replace q13_5_medical_aid=. if q13_5_medical_aid==2

recode q13_5_medical_aid (0=1) (1=2)

ta q13_1_healthcare [iw=DOWNSCALE_MUN_PP_BENCHWGT]

replace q13_1_healthcare=. if q13_1_healthcare>=4
```

```
*Household-level factors

*HH Wealth index

*ta q14_8_head [iw=DOWNSCALE_MUN_PP_BENCHWGT]

ta q14_5_people_recode [iw=DOWNSCALE_MUN_PP_BENCHWGT]

recode q14_5_people_recode (1=1 "One") (2=2 "Two") (3=3 "Three") (4/7=4 "Four_More"), gen
(People_in_HH)

ta People_in_HH [iw=DOWNSCALE_MUN_PP_BENCHWGT]

ta q14_6_under18_recode [iw=DOWNSCALE_MUN_PP_BENCHWGT]

ta q14_7_60plus_recode [iw=DOWNSCALE_MUN_PP_BENCHWGT]

ta q6_5_feed_children [iw=DOWNSCALE_MUN_PP_BENCHWGT]

recode q6_5_feed_children (0=1) (1=2) (2=3)

ta q14_10_social_grant [iw=DOWNSCALE_MUN_PP_BENCHWGT]

recode q14_10_social_grant (0=1) (1=2)


*Community-level factors

gen Media=.

replace Media= 1 if q6_3_1_landline==0 & q6_3_2_cell==0 & q6_3_3_tv==0 &
q6_3_4_computer==0 & q6_3_5_radio==0 & q6_3_6_dstv==0 & q6_3_7_internet==0

replace Media= 2 if q6_3_1_landline==1 & q6_3_2_cell==1 & q6_3_3_tv==1 &
q6_3_4_computer==1 & q6_3_5_radio==1 & q6_3_6_dstv==1 & q6_3_7_internet==1

recode Media (1=1 "No") (2=2 "Yes"), gen (Media_Access)

ta Media_Access [iw=DOWNSCALE_MUN_PP_BENCHWGT]

*q6_1_internet



*Residential status

ta a3_dwelling_type_recode [iw=DOWNSCALE_MUN_PP_BENCHWGT]

replace a3_dwelling_type_recode=. if a3_dwelling_type_recode==3



*Individual factors


ta Migration_Status

gen life_sati_5=Life_Satisfaction if Migration_Status==1 | Migration_Status==2
```

```
gen lifesatisfaction=LifeSati if Migration_Status==1 | Migration_Status==2
gen groupedage=Grouped_Age if Migration_Status==1 | Migration_Status==2
gen sex=a2_sex if Migration_Status==1 | Migration_Status==2
gen highesteducation=Highest_Education if Migration_Status==1 | Migration_Status==2
gen populationgroup=Population_group if Migration_Status==1 | Migration_Status==2
gen income=Income if Migration_Status==1 | Migration_Status==2
gen working=q10_2_working if Migration_Status==1 | Migration_Status==2
gen marritalstatus=Marrital_Status if Migration_Status==1 | Migration_Status==2
gen medicalaid=q13_5_medical_aid if Migration_Status==1 | Migration_Status==2
gen healthfacility=q13_1_healthcare if Migration_Status==1 | Migration_Status==2

*Household-level factors
gen hhhead=q14_8_head if Migration_Status==1 | Migration_Status==2
recode hhhead (1/9=1 "HhH"), gen (HHead)
ta HHead
gen HHeadSex=.
replace HHeadSex=1 if HHead==1 & sex==1
replace HHeadSex=2 if HHead==1 & sex==2
ta HHeadSex [iw=DOWNSCALE_MUN_PP_BENCHWGT]
gen hhmembers=People_in_HH if Migration_Status==1 | Migration_Status==2
gen under18=q14_6_under18_recode if Migration_Status==1 | Migration_Status==2
gen sixtyplus=q14_7_60plus_recode if Migration_Status==1 | Migration_Status==2
gen childhunger=q6_5_feed_children if Migration_Status==1 | Migration_Status==2
gen social_grant=q14_10_social_grant if Migration_Status==1 | Migration_Status==2

*Community-level factors
gen dwellingtype=a3_dwelling_type_recode if Migration_Status==1 | Migration_Status==2
*gen Migration_Status if Migration_Status==1 | Migration_Status==2
gen mediaaccess=Media_Access if Migration_Status==1 | Migration_Status==2


drop if Migration_Status==.
drop if lifesatisfaction==.
drop if groupedage==.
```

```
drop if sex==.
drop if highesteducation==.
drop if populationgroup==.
drop if income==.
drop if working==.
drop if marritalstatus==.
drop if medicalaid==.
drop if healthfacility==.
drop if dwellingtype==.

*Frequency by migration status
ta life_sati_5 Migration_Status [iw=DOWNSCALE_MUN_PP_BENCHWGT]
ta Migration_Status [iw=DOWNSCALE_MUN_PP_BENCHWGT]
ta lifesatisfaction Migration_Status [iw=DOWNSCALE_MUN_PP_BENCHWGT]
ta lifesatisfaction sex [iw=DOWNSCALE_MUN_PP_BENCHWGT]
ta groupedage Migration_Status [iw=DOWNSCALE_MUN_PP_BENCHWGT]
ta sex Migration_Status [iw=DOWNSCALE_MUN_PP_BENCHWGT]
ta highesteducation Migration_Status [iw=DOWNSCALE_MUN_PP_BENCHWGT]
ta populationgroup Migration_Status [iw=DOWNSCALE_MUN_PP_BENCHWGT]
ta income Migration_Status [iw=DOWNSCALE_MUN_PP_BENCHWGT]
ta working Migration_Status [iw=DOWNSCALE_MUN_PP_BENCHWGT]
ta marritalstatus Migration_Status [iw=DOWNSCALE_MUN_PP_BENCHWGT]
ta medicalaid Migration_Status [iw=DOWNSCALE_MUN_PP_BENCHWGT]
ta healthfacility Migration_Status [iw=DOWNSCALE_MUN_PP_BENCHWGT]

*Household-level factors
ta HHeadSex Migration_Status [iw=DOWNSCALE_MUN_PP_BENCHWGT]
ta hhmembers Migration_Status [iw=DOWNSCALE_MUN_PP_BENCHWGT]
ta under18 Migration_Status [iw=DOWNSCALE_MUN_PP_BENCHWGT]
ta sixtyplus Migration_Status [iw=DOWNSCALE_MUN_PP_BENCHWGT]
ta childhunger Migration_Status [iw=DOWNSCALE_MUN_PP_BENCHWGT]
ta social_grant Migration_Status [iw=DOWNSCALE_MUN_PP_BENCHWGT]

*Community-level factors
```

ta dwellingtype Migration_Status [iw=DOWNSCALE_MUN_PP_BENCHWGT]

ta Migration_Status Migration_Status [iw=DOWNSCALE_MUN_PP_BENCHWGT]

ta mediaaccess Migration_Status [iw=DOWNSCALE_MUN_PP_BENCHWGT]

*Frequency by sex

ta Migration_Status [iw=DOWNSCALE_MUN_PP_BENCHWGT]

ta life_sati_5 Migration_Status [iw=DOWNSCALE_MUN_PP_BENCHWGT]

ta lifesatisfaction [iw=DOWNSCALE_MUN_PP_BENCHWGT]

ta lifesatisfaction Migration_Status [iw=DOWNSCALE_MUN_PP_BENCHWGT]

ta lifesatisfaction sex [iw=DOWNSCALE_MUN_PP_BENCHWGT]

ta groupedage sex [iw=DOWNSCALE_MUN_PP_BENCHWGT]

ta sex Migration_Status [iw=DOWNSCALE_MUN_PP_BENCHWGT]

ta highesteducation sex [iw=DOWNSCALE_MUN_PP_BENCHWGT]

ta populationgroup sex [iw=DOWNSCALE_MUN_PP_BENCHWGT]

ta income sex [iw=DOWNSCALE_MUN_PP_BENCHWGT]

ta working sex [iw=DOWNSCALE_MUN_PP_BENCHWGT]

ta marritalstatus sex [iw=DOWNSCALE_MUN_PP_BENCHWGT]

ta medicalaid sex [iw=DOWNSCALE_MUN_PP_BENCHWGT]

ta healthfacility sex [iw=DOWNSCALE_MUN_PP_BENCHWGT]

*Household-level factors

ta HHeadSex [iw=DOWNSCALE_MUN_PP_BENCHWGT]

ta hhmembers sex [iw=DOWNSCALE_MUN_PP_BENCHWGT]

ta under18 sex [iw=DOWNSCALE_MUN_PP_BENCHWGT]

ta sixtyplus sex [iw=DOWNSCALE_MUN_PP_BENCHWGT]

ta childhunger sex [iw=DOWNSCALE_MUN_PP_BENCHWGT]

ta social_grant sex [iw=DOWNSCALE_MUN_PP_BENCHWGT]

*Community-level factors

ta dwellingtype sex [iw=DOWNSCALE_MUN_PP_BENCHWGT]

ta Migration_Status sex [iw=DOWNSCALE_MUN_PP_BENCHWGT]

ta mediaaccess sex [iw=DOWNSCALE_MUN_PP_BENCHWGT]

```
******************************************
table sex life_sati_5 Migration_Status [iw=DOWNSCALE_MUN_PP_BENCHWGT]
table life_sati_5 Migration_Status [iw=DOWNSCALE_MUN_PP_BENCHWGT]
```
