# Supplementary material for: Determinants of life satisfaction among migrants in South Africa: an analysis of the GCRO’s quality of life survey (2009–2021)
Source: BMC Public Health. 2023 Oct 18;23:2030. doi: 10.1186/s12889-023-16868-1 (PMC10585904; doi:10.1186/s12889-023-16868-1)
Supplement: Supplementary file 9 — Supplementary Material 9 [file 12889_2023_16868_MOESM9_ESM.pdf]

```
*Life satisfaction

clear

use "C:Merged data\2019 2020.dta", clear

merge m:m Unique_Identifier ward WWeight Migration_Status life_sati_5 life_sati_5 groupedage sex highesteducation populationgroup income working medicalaid healthfacility HHeadSex hhmembers under18 childhunger social_grant dwellingtype mediaaccess using "C:\Users\Professor Likoko S\Desktop\Short data\2017 2018.dta"

drop _merge

merge m:m Unique_Identifier ward WWeight Migration_Status life_sati_5 life_sati_5 groupedage sex highesteducation populationgroup income working medicalaid healthfacility HHeadSex hhmembers under18 childhunger social_grant dwellingtype mediaaccess using "C:\Users\Professor Likoko S\Desktop\Short data\2015 2016.dta"

drop _merge

merge m:m Unique_Identifier ward WWeight Migration_Status life_sati_5 life_sati_5 groupedage sex highesteducation populationgroup income working medicalaid healthfacility HHeadSex hhmembers under18 childhunger social_grant dwellingtype mediaaccess using "C:\Users\Professor Likoko S\Desktop\Short data\2013 2014.dta"

drop _merge

merge m:m Unique_Identifier ward WWeight Migration_Status life_sati_5 life_sati_5 groupedage sex highesteducation populationgroup income working medicalaid healthfacility HHeadSex hhmembers under18 childhunger social_grant dwellingtype mediaaccess using "C:\Users\Professor Likoko S\Desktop\Short data\2011.dta"

drop _merge

merge m:m Unique_Identifier ward WWeight Migration_Status life_sati_5 life_sati_5 groupedage sex highesteducation populationgroup income working medicalaid healthfacility HHeadSex hhmembers under18 childhunger social_grant dwellingtype mediaaccess using "C:\Users\Professor Likoko S\Desktop\Short data\2009.dta"


drop if Migration_Status==.

drop if life_sati_5==.

drop if groupedage==.

drop if sex==.

drop if highesteducation==.

drop if populationgroup==.

drop if income==.

drop if working==.
```

```
drop if medicalaid==.
drop if healthfacility==.
drop if dwellingtype==.
drop if HHeadSex==.
drop if hhmembers==.
drop if under18==.
drop if childhunger==.
drop if social_grant==.


*******************************************************************************


*Frequency by migration status
ta life_sati_5 [iw=WWeight]
ta life_sati_5 Migration_Status [iw=WWeight]
ta life_sati_5 sex if Migration_Status==1 | Migration_Status==2 [iw=WWeight]
ta Migration_Status [iw=WWeight]
*ta lifesatisfaction Migration_Status [iw=WWeight]
*ta lifesatisfaction sex [iw=WWeight]
ta groupedage Migration_Status [iw=WWeight]
ta sex Migration_Status [iw=WWeight]
ta highesteducation Migration_Status [iw=WWeight]
ta populationgroup Migration_Status [iw=WWeight]
ta income Migration_Status [iw=WWeight]
ta working Migration_Status [iw=WWeight]
*ta marritalstatus Migration_Status [iw=WWeight]
ta medicalaid Migration_Status [iw=WWeight]
ta healthfacility Migration_Status [iw=WWeight]

*Household-level factors
ta HHeadSex Migration_Status [iw=WWeight]
ta hhmembers Migration_Status [iw=WWeight]
ta under18 Migration_Status [iw=WWeight]
```

```
*ta sixtyplus Migration_Status [iw=WWeight]
ta childhunger Migration_Status [iw=WWeight]
ta social_grant Migration_Status [iw=WWeight]

*Community-level factors
ta dwellingtype Migration_Status [iw=WWeight]
ta Migration_Status Migration_Status [iw=WWeight]
*ta mediaaccess Migration_Status [iw=WWeight]


*Frequency by sex
ta Migration_Status [iw=WWeight]
ta life_sati_5 [iw=WWeight]
ta life_sati_5 Migration_Status [iw=WWeight]
ta life_sati_5 sex [iw=WWeight]
ta groupedage sex [iw=WWeight]
ta sex [iw=WWeight]
ta highesteducation sex [iw=WWeight]
ta populationgroup sex [iw=WWeight]
ta income sex [iw=WWeight]
ta working sex [iw=WWeight]
*ta marritalstatus sex [iw=WWeight]
ta medicalaid sex [iw=WWeight]
ta healthfacility sex [iw=WWeight]

*Household-level factors
*ta HHeadSex sex [iw=WWeight]
ta hhmembers sex [iw=WWeight]
ta under18 sex [iw=WWeight]
*ta sixtyplus sex [iw=WWeight]
ta childhunger sex [iw=WWeight]
ta social_grant sex [iw=WWeight]

*Community-level factors
```

```
ta dwellingtype sex [iw=WWeight]
ta Migration_Status sex [iw=WWeight]
ta mediaaccess sex [iw=WWeight]

******************************************
table sex life_sati_5 Migration_Status [iw=WWeight]
table life_sati_5 Migration_Status [iw=WWeight]


*In-migrants
*Migration_Status Migration_Status
gen Ilife_sati_5 = life_sati_5 if Migration_Status==1
gen Isex = sex  if Migration_Status==1
gen Igroupedage = groupedage if Migration_Status==1
gen Ihighesteducation = highesteducation  if Migration_Status==1
gen Ipopulationgroup = populationgroup if Migration_Status==1
gen Iincome = income  if Migration_Status==1
gen Iworking = working if Migration_Status==1
gen Imedicalaid = medicalaid  if Migration_Status==1
gen Ihealthfacility = healthfacility  if Migration_Status==1
gen IHHeadSex = HHeadSex if Migration_Status==1
gen Ihhmembers = hhmembers  if Migration_Status==1
gen Iunder18 = under18  if Migration_Status==1
gen Ichildhunger = childhunger  if Migration_Status==1
gen Isocial_grant = social_grant if Migration_Status==1
gen Idwellingtype = dwellingtype  if Migration_Status==1
gen Imediaaccess = mediaaccess if Migration_Status==1

*Immigrants
gen Imlife_sati_5 = life_sati_5 if Migration_Status==2
gen Imsex = sex  if Migration_Status==2
gen Imgroupedage = groupedage if Migration_Status==2
gen Imhighesteducation = highesteducation  if Migration_Status==2
gen Impopulationgroup = populationgroup if Migration_Status==2
```

```
gen Imincome = income  if Migration_Status==2
gen Imworking = working if Migration_Status==2
gen Immedicalaid = medicalaid  if Migration_Status==2
gen Imhealthfacility = healthfacility  if Migration_Status==2
gen ImHHeadSex = HHeadSex if Migration_Status==2
gen Imhhmembers = hhmembers  if Migration_Status==2
gen Imunder18 = under18  if Migration_Status==2
gen Imchildhunger = childhunger  if Migration_Status==2
gen Imsocial_grant = social_grant if Migration_Status==2
gen Imdwellingtype = dwellingtype  if Migration_Status==2
gen Immediaaccess = mediaaccess if Migration_Status==2

*Pearson's Product Moment Correlation Coefficient
*pwcorr VariableA VariableB, sig star(.05) obs

*In-migrants
pwcorr Isex Ilife_sati_5, sig star(.05) obs
pwcorr Igroupedage Ilife_sati_5, sig star(.05) obs
pwcorr Ihighesteducation Ilife_sati_5, sig star(.05) obs
pwcorr Ipopulationgroup Ilife_sati_5, sig star(.05) obs
pwcorr Iincome Ilife_sati_5, sig star(.05) obs
pwcorr Iworking Ilife_sati_5, sig star(.05) obs
pwcorr Imedicalaid Ilife_sati_5, sig star(.05) obs
pwcorr Ihealthfacility Ilife_sati_5, sig star(.05) obs
pwcorr IHHeadSex Ilife_sati_5, sig star(.05) obs
pwcorr Ihhmembers Ilife_sati_5, sig star(.05) obs
pwcorr Iunder18 Ilife_sati_5, sig star(.05) obs
pwcorr Ichildhunger Ilife_sati_5, sig star(.05) obs
pwcorr Isocial_grant Ilife_sati_5, sig star(.05) obs
pwcorr Idwellingtype Ilife_sati_5, sig star(.05) obs
pwcorr Imediaaccess Ilife_sati_5, sig star(.05) obs

*Immigrants
pwcorr Imsex Imlife_sati_5, sig star(.05) obs
```

```
pwcorr Imgroupedage Imlife_sati_5, sig star(.05) obs
pwcorr Imhighesteducation Imlife_sati_5, sig star(.05) obs
pwcorr Impopulationgroup Imlife_sati_5, sig star(.05) obs
pwcorr Imincome Imlife_sati_5, sig star(.05) obs
pwcorr Imworking Imlife_sati_5, sig star(.05) obs
pwcorr Immedicalaid Imlife_sati_5, sig star(.05) obs
pwcorr Imhealthfacility Imlife_sati_5, sig star(.05) obs
pwcorr ImHHeadSex Imlife_sati_5, sig star(.05) obs
pwcorr Imhhmembers Imlife_sati_5, sig star(.05) obs
pwcorr Imunder18 Imlife_sati_5, sig star(.05) obs
pwcorr Imchildhunger Imlife_sati_5, sig star(.05) obs
pwcorr Imsocial_grant Imlife_sati_5, sig star(.05) obs
pwcorr Imdwellingtype Imlife_sati_5, sig star(.05) obs
pwcorr Immediaaccess Imlife_sati_5, sig star(.05) obs


*In-migrants
*Life_sat sex
gen MIlife_sati_5 = life_sati_5 if sex==1
gen MIsex = sex  if sex==1
gen MIgroupedage = groupedage if sex==1
gen MIhighesteducation = highesteducation  if sex==1
gen MIpopulationgroup = populationgroup if sex==1
gen MIincome = income  if sex==1
gen MIworking = working if sex==1
gen MImedicalaid = medicalaid  if sex==1
gen MIhealthfacility = healthfacility  if sex==1
gen MIHHeadSex = HHeadSex if sex==1
gen MIhhmembers = hhmembers  if sex==1
gen MIunder18 = under18  if sex==1
gen MIchildhunger = childhunger  if sex==1
gen MIsocial_grant = social_grant if sex==1
gen MIdwellingtype = dwellingtype  if sex==1
gen MImediaaccess = mediaaccess if sex==1
```

gen MIMigration_Status = Migration_Status if sex==1

*Immigrants

*Life_sat sex

gen FIlife_sati_5 = life_sati_5 if sex==2

gen FIsex = sex if sex==2

gen FIgroupedage = groupedage if sex==2

gen FIhighesteducation = highesteducation if sex==2

gen FIpopulationgroup = populationgroup if sex==2

gen FIincome = income if sex==2

gen FIworking = working if sex==2

gen FImedicalaid = medicalaid if sex==2

gen FIhealthfacility = healthfacility if sex==2

gen FIHHeadSex = HHeadSex if sex==2

gen FIhhmembers = hhmembers if sex==2

gen FIunder18 = under18 if sex==2

gen FIchildhunger = childhunger if sex==2

gen FIsocial_grant = social_grant if sex==2

gen FIdwellingtype = dwellingtype if sex==2

gen FImediaaccess = mediaaccess if sex==2

gen FIMigration_Status = Migration_Status if sex==2

oprobit MIlife_sati_5 i.MIgroupedage i.MIhighesteducation i.MIpopulationgroup i.MIincome i.MIworking i.MImedicalaid i.MIhealthfacility i.MIhhmembers i.MIunder18 i.MIchildhunger i.MIsocial_grant i.MIdwellingtype i.MIMigration_Status, nolog

oprobit FIlife_sati_5 i.FIgroupedage i.FIhighesteducation i.FIpopulationgroup i.FIincome i.FIworking i.FImedicalaid i.FIhealthfacility i.FIhhmembers i.FIunder18 i.FIchildhunger i.FIsocial_grant i.FIdwellingtype i.FIMigration_Status, nolog

oprobit life_sati_5 i.sex i.groupedage i.highesteducation i.populationgroup i.income i.working i.medicalaid i.healthfacility i.hhmembers i.under18 i.childhunger i.social_grant i.dwellingtype i.Migration_Status, nolog

ologit life_sati_5 i.sex i.groupedage i.highesteducation i.populationgroup i.income i.working i.medicalaid i.healthfacility i.hhmembers i.under18 i.childhunger i.social_grant i.dwellingtype i.Migration_Status, nolog

margins MIgroupedage MIhighesteducation MIpopulationgroup MIincome MIworking MImedicalaid MIhealthfacility MIhhmembers MIunder18 MIchildhunger MIsocial_grant MIdwellingtype MIMigration_Status

margins FIgroupedage FIhighesteducation FIpopulationgroup FIincome FIworking FImedicalaid FIhealthfacility FIhhmembers FIunder18 FIchildhunger FIsocial_grant FIdwellingtype FIMigration_Status
